# *MiningABs*: mining associated biomarkers across multi-connected gene expression datasets

**DOI:** 10.1186/1471-2105-15-173

**Published:** 2014-06-08

**Authors:** Chun-Pei Cheng, Christopher DeBoever, Kelly A Frazer, Yu-Cheng Liu, Vincent S Tseng

**Affiliations:** 1 Department of Computer Science and Information Engineering, National Cheng Kung University, Tainan, Taiwan; 2 Moores UCSD Cancer Center, University of California San Diego, La Jolla, California, USA; 3 Department of Pediatrics and Rady Children’s Hospital, University of California San Diego, La Jolla, California, USA; 4 Institute for Genomic Medicine, University of California San Diego, La Jolla, California, USA; 5 Department of Environmental and Occupational Health, National Cheng Kung University, Tainan, Taiwan; 6 Institute of Medical Informatics, National Cheng Kung University, Tainan, Taiwan

**Keywords:** Mining ABs, Associated biomarkers, Meta-analysis, Combination effects, Gene expression

## Abstract

**Background:**

Human disease often arises as a consequence of alterations in a set of associated genes rather than alterations to a set of unassociated individual genes. Most previous microarray-based meta-analyses identified disease-associated genes or biomarkers independent of genetic interactions. Therefore, in this study, we present the first meta-analysis method capable of taking gene combination effects into account to efficiently identify *associated biomarkers* (*ABs*) across different microarray platforms.

**Results:**

We propose a new meta-analysis approach called *MiningABs* to mine *ABs* across different array-based datasets. The similarity between paired *probe* sequences is quantified as a bridge to connect these datasets together. The *ABs* can be subsequently identified from an “improved” *common logit model* (*c-LM*) by combining several *sibling-like LMs* in a heuristic genetic algorithm selection process. Our approach is evaluated with two sets of gene expression datasets: i) 4 esophageal squamous cell carcinoma and ii) 3 hepatocellular carcinoma datasets. Based on an unbiased reciprocal test, we demonstrate that each gene in a group of *ABs* is required to maintain high cancer sample classification accuracy, and we observe that *ABs* are not limited to genes common to all platforms. Investigating the *ABs* using Gene Ontology (GO) enrichment, literature survey, and network analyses indicated that our *ABs* are not only strongly related to cancer development but also highly connected in a diverse network of biological interactions.

**Conclusions:**

The proposed meta-analysis method called *MiningABs* is able to efficiently identify *ABs* from different independently performed array-based datasets, and we show its validity in cancer biology via GO enrichment, literature survey and network analyses. We postulate that the *ABs* may facilitate novel target and drug discovery, leading to improved clinical treatment. Java source code, tutorial, example and related materials are available at “http://sourceforge.net/projects/miningabs/”.

## Background

Many clinical diseases such as cancer arise as a consequence of massive alterations in gene activity. Genes may interact or work together in response to environmental change, further influencing the fate of a cell [1]. Synthetic lethality, where cell death is observed when two genes are mutated but not when only one of the pair is mutated, is a classic example of genetic interaction [2]. Another example is in causal SNP identification where Han *et al.* showed that two associated SNPs in the non-coding region of *CFH* (complement factor H) were linked to age-related macular degeneration [3]. In our previous studies, we also demonstrated that co-expressed genes revealed from association rules are associated in yeast cells when they suffered from different stresses [4]. Therefore, many lines of evidence suggest that combination effects of certain genes influence biological outcomes rather than individual effects of a set of unassociated individual genes.

In the past decade, microarray techniques have been widely used to detect large-scale molecular changes in many biological events such as alterations in gene expression for human tumorigenesis [5-9]. These approaches identified some important cancer-associated genes and cellular pathways. However, most of these discoveries were made using statistical methods such as applying a principal component analysis to obtain a limited gene list or using the *t-*test to determine whether any *probe* readings were significantly different between matched normal and tumor samples [7]. Here, a *probe* reading is defined as the final intensity of a cell-isolated nucleotide sequence hybridized to a probe set containing 25 bp probe sequences derived from a genomic target region of a gene in the Affymetrix array platform or hybridized to a 60 bp spotted sequence of a gene in the Agilent array platform. Despite similar experimental and analytical designs, the results of these studies often have little or no overlap [5,7]. These results motivated others to develop meta-analysis methods to discover reliable common patterns across different individually performed experiments.

Existing microarray meta-analysis methods, reviewed recently by Dr. George C. Tseng and his colleague [10,11], use a variety of strategies including i) vote counting, ii) combining p-values, iii) combining effect sizes, iv) combining ranks and v) directly merging after normalization. The vote counting method counts how many curated independent datasets show significant gene expression changes between paired case–control samples for a queried gene. For example, LaCroix-Fralish *et al.* selected 79 pain-related genes to be statistically significant “hits” in 4 or more independent experiments using the vote counting-based binomial test and then confirmed 43 out of the 79 using qPCR in the dorsal root ganglion of rat with chronic constriction injury [12]. Although this method is very straightforward and efficient to find candidate genes common to different experiments, the method relies highly on the definition of significance used in the original researches. Considering more quantitative information like *p*-values or even fold changes of genes between two groups of samples might help increase the flexibility and utility of meta-analysis. Rhodes *et al.* integrated one-sided permutation t-test *p*-values for each gene that is present in all collected prostate cancer gene expression profiles [13]. Similarly, combining single study-derived *p*-values or transform scores for two- [14,15] and multi-class [16] comparisons has also been conducted in the previous literature. Regarding the approach using effect size, Choi *et al.* integrated *t*-based effect size (fold-change in gene expression) to discover significant genes from cancer datasets [17], and Wang *et al.* utilized Bayesian statistics to identify differentially expressed genes between B-cell chronic lymphocytic leukemia and normal B cells across three microarray studies [18]. However, using the combination of either p-values or effect sizes, it is likely to obtain many candidate differentially expressed genes that are outliers actually. Incorporating rank statistics of genes in the aforementioned p-values or effect sizes in each study might help fix this problem. For this, Hong *et al.* successfully proposed a non-parametric fold-change-to-rank statistic to detect plant hormone-related genes [19], and Sanford *et al.* applied it to sub-classify renal neoplasms [20]. In addition to the above reviewed meta-analyses, recently there are some newer sophisticated methods like following the PRISMA statement [21] to calculate Cochran’s Q statistic [22] for each gene across datasets curated in the study, or identifying genes by directly merging data sets after normalizing the data [23]. Although the above methods have been developed and evaluated with different sources of gene expression microarrays, the resulting genes were still considered independently associated with their target diseases. Discovering associated significant genes across different microarray datasets, so called *associated biomarkers* (*ABs*), is a novel approach for identifying convincing mechanisms underlying biological events or new targets for drug design.

In this study, we propose a new method called *MiningABs* to discover *ABs* through an “improved” *common logit model* (*c-LM*) discovered from multiple connected datasets. The *logit model* (*LM*) is a useful method for solving binary classification problems such as classification of samples as tumor or normal. The *LM* states that the probability of belonging to a clinical group can be formulated as a function of differences in gene expression. *MiningABs* attempts to find a small subset of genes, the *ABs*, that have a high classification accuracy under the *LM.* We use a heuristic genetic algorithm to select variables for the *LM* that allows for an optimal model to be discovered in a reasonable time period. Genetic algorithms have been used to select pathological variables to predict myocardial infarction [24] and radiotherapy treatment outcomes [25] using *LMs*. While this approach is very powerful for most optimization problems, previous studies were limited to a single data set or a single experiment. The challenges of using genetic algorithms to select variables in a *LM* with different microarray datasets include: i) how to handle the input platforms containing disparate number of *probes* and genes, ii) how to efficiently discover *ABs* from any possible gene combinations other than a brute force search, iii) how to evaluate whether the identified *ABs* are relevant to a biological event, and iv) what number of *ABs* provides the best classification accuracy. Our method addresses all of these issues and is evaluated with two publicly available cancer microarray datasets: i) 4 gene expression microarray datasets conducted by 3 independent research groups in human esophageal squamous cell carcinoma [5-7] and ii) 3 gene expression microarray datasets in human hepatocellular carcinoma [8,9].

## Methods

### Overview of datasets

In this study, two input sets of gene expression microarray data for human cancer subjects, esophageal squamous cell carcinoma (ESCC) and hepatocellular carcinoma (HCC), were accessed from the Gene Expression Omnibus (GEO) database. Table 1 shows the detailed characteristics of these datasets. Four ESCC [5-7] and 3 HCC [8,9] independent experimental designs were conducted to identify differentially expressed genes of interest using various microarray platforms and clinical samples. Su *et al.* performed global gene expression profiling and validation to identify 7 ESCC-related genes and their associations with clinical phenotypes. Hu *et al.* identified 12 ESCC-related genes relevant to DNA copy number neutral loss of heterozygosity, and Yan *et al.* also identified 12 putative therapeutic targets/genes in ESCC treatment. For the input HCC set, Roessler *et al.* provided two large-scale microarray datasets and identified 6 human chromosome 8p-invovled genes associated with HCC and patient survival, and finally Tsuchiya *et al.* identified 11 HCC-related genes from hepatitis C virus-positive patients. However, esophageal carcinoma is the 8^th^ most common cancer worldwide affecting more than 450,000 patients annually, and it is the 6^th^ leading cause of cancer-related mortality with more than 400,000 deaths per year [26,27]. Additionally, hepatocellular carcinoma is the 6^th^ most common cancer worldwide and the 3^rd^ most common cause of cancer-related death [28]. Hence, mining more cancer-related patterns in gene expression will help us identify more key genes involved in these diseases and provide more information for developing therapeutics. The two input sets will be individually considered as the inputs to our method. The detailed data processing steps will be introduced in the following paragraphs.

**Table 1:** Characteristics of microarray datasets used in this study

**Sample types**	**Dataset serial numbers**	**GEO accession numbers**	**Platform types**	**N/T**	**A/D**	**# of distinct genes in a platform**	**Avg length of sequences**	**Source of samples**	**References**
							**(Avg ± SD)**		
ESCC	1-1	GSE23400	Affymetrix HG-U133A	53/53	20,133/22,283	12,633	250 ± 22^†^	China	[5]
	1-2	GSE23400	Affymetrix HG-U133B	51/51	14,110/22,477	9,256	250 ± 22^†^		
	1-3	GSE20347	Affymetrix HG-U133A_2	17/17	20,133/22,277	12,633	250 ± 22^†^	China	[6]
	1-4	GSE29001	Affymetrix HG-U133A_2	12/12	20,133/22,277	12,633	250 ± 22^†^	China	[7]
HCC	2-1	GSE14520	Affymetrix HG-U133A_2	19/22	20,133/22,277	12,633	250 ± 22^†^	China	[8]
	2-2	GSE14520	Affymetrix HT_HG-U133A	210/225	20,429/22,277	12,743	440 ± 105^†^		
	2-3	GSE17856	Agilent 014850	44/43	20,772/25,073	14,312	60 ± 0^††^	Japan	[9]

ESCC: esophageal squamous cell carcinoma; HCC: hepatocellular carcinoma; N: # of normal samples; T: # of tumor samples; A: # of available *probes* matched with distinguishable gene IDs in a platform; D: # of downloaded *probes* contained in a platform; Avg: average; SD: standard deviation; ^†^: Affymetrix probe set-matched target sequence; ^††^: Agilent spotted sequence.

### Integrating *sk-LMs* to classify cancer samples

In this section, we introduce how to discover the *associated biomarkers* (*ABs*) for a *common logit model* (*c-LM*) by combining *sibling-like logit models* (*sk-LMs*) derived separately for each dataset. The basic definition of the traditional *LM* developed from a single microarray dataset will be given in the first part. Then, we introduce how to link different datasets together with a matrix of *probe* sequence (including Affymetrix probe set-matched target sequence and Agilent spotted sequence) similarities, and finally introduce how to determine a *c-LM* from the multi-connected-datasets.

#### *Development of individual logit model from single dataset*

The traditional *logit model* (*LM*) is a commonly used method for solving binary classification problems and is akin to non-linear regression such as fitting a polynomial to a set of numerical/categorical data. In this case, the probability *p* of a sample being from a cancer patient is derived as a function of the following combination of *n* selected *probe* readings *x* = {*x*_*1*_, *x*_*2*_,…, *x*_*n*_}. A general form of the *LM* formula is given by Eq. (1).

(1) $$p=\frac{e^{\beta_{0}+\beta_{1}x_{1}+\beta_{2}x_{2}+\ldots +\beta_{n}x_{n}}}{1+e^{\beta_{0}+\beta_{1}x_{1}+\beta_{2}x_{2}+\ldots +\beta_{n}x_{n}}}$$

where *β*_*0*_ is an intercept and {*β*_*1*_, *β*_*2*_,…, *β*_*n*_} are coefficients of the independent variables. If the target categorical variable is tumor, *p* will be set as 1. On the contrary, normal samples are set as 0. We use a maximum likelihood estimation method to calculate these beta values. The *LM* for a single dataset can be evaluated by referring to the natural log likelihood value (*LLV*) via the following Eq. (2).

(2) LLVβ^=ln∏i=1nπ^xiyi1-π^xi1-yi

π^xi=eβ^0+β^1xi1+β^2xi2+…+β^nxin1+eβ^0+β^1xi1+β^2xi2+…+β^nxin

Where β^ are the beta values of a *LM* and *y*_*i*_ is the target categorical value (Tumor = 1 and Normal = 0) of the indexed *i* out of *n* samples. The domain of *LLV* is less than zero, and a larger *LLV* represents a better model in sample fitness.

However, developing an individual *LM* from a single array dataset may not offer maximum power for classifying cancer samples because the number of available *probes*/genes varies from platform to platform (Table 1). As a result, a single array dataset-derived classification model may not be applicable for another dataset. In most of the traditional meta-analysis approaches, the common significant genes are limited to the genes common to all microarray platforms [13,18]. Table 1 shows that among the available *probes*, the numbers of common genes across input ESCC and HCC sets are only 3,887 and 9,755 respectively. Many potential candidate genes would be missed if an approach only focuses on these common genes. Therefore, developing a *c-LM* in which every *AB*’s value can be accessed simultaneously in each dataset is a very important issue. In the next section, we introduce how to overcome this issue by linking different array-based platforms together using a sequence comparison-based method.

#### *Probe sequence similarity matrix development as a bridge to connect datasets*

**Figure 1:**
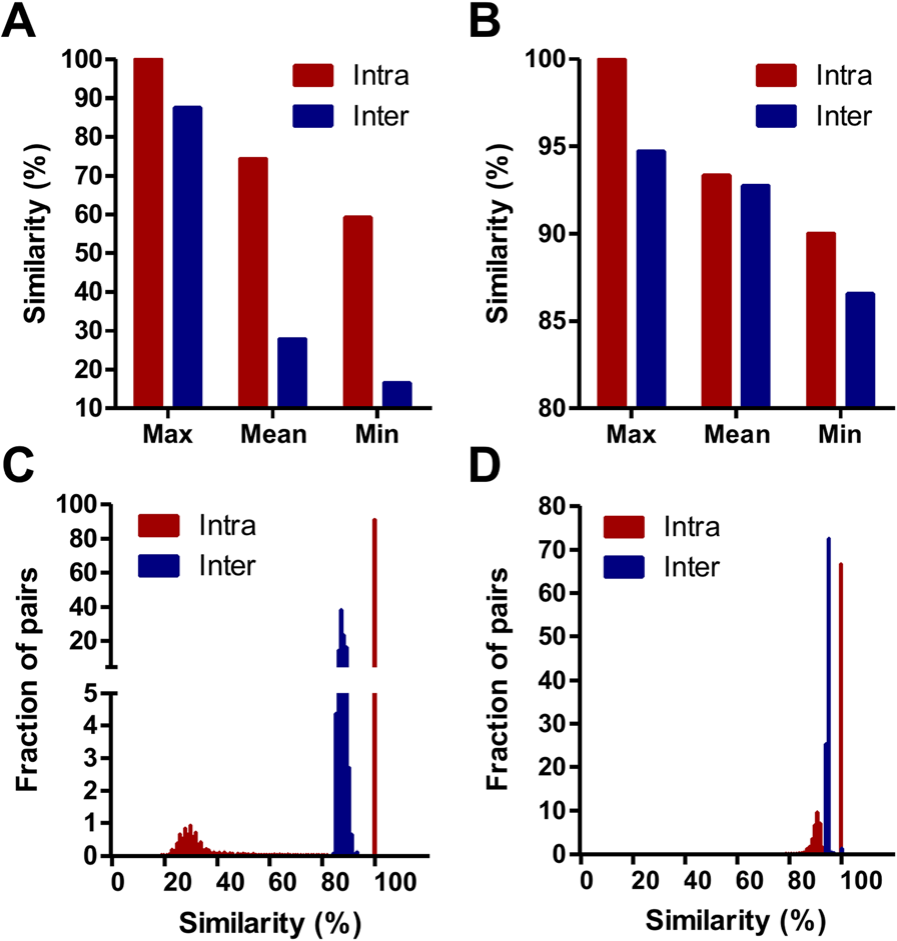
**Comparisons of paired*probe*sequences in two input sets. A)** Average of maximum/mean/minimum similarity scores among different *probe* sequence sets in ESCC set. **B)** Average similarity as a function of maximum/average/minimum scores among different *probe* sequence sets in HCC set. **C)** Distributions of most similar paired *probe* sequences (Max group in the panel **A**). **D)** Distributions of most similar paired *probe* sequences (Max group in the panel **B**). Intra: different *probes* matched with same gene IDs; Inter: different *probes* matched with different gene IDs.

The final *probe* readings from arrays are only based on the sequence-sequence hybridization affinities. Although *probes* are matched with different gene IDs over array platforms, the readings will be very similar for a given cell-isolated nucleotide sequence. Moreover, the hybridized sequences are usually limited to a very short sub-sequence (hundred bases) of gene open reading frames rather than the entire gene. Is it possible to designate a *probe* as a substitute for a *probe* that is contained by one platform but not another by finding *probes* with the highest similarity in sequences between the two platforms? To test this possibility, we measured a similarity score for each paired *probe* sequence in both input platforms. The Affymetrix probe set-matched target sequence (the sequence from which 25 bp sequences within probe sets are extracted, average length 250 bp or 440 bp) and Agilent spotted sequences (average length 60 bp) were used in this study (Table 1). In Figure 1A and B, the averages of maximum/mean/minimum similarity scores among the *probe* sequences for the same gene (intra) are higher than *probes* from different genes (inter). In the intra group, 91% (Figure 1C) and 67% (Figure 1D) of the *probes* can be matched to at least one different *probe* on another platform. *Probes* from different genes can often be matched with a most similar (above 80%) *probe* in the ESCC (Figure 1C) and HCC (Figure 1D) input sets. These observations hinted that using a most similar substitute in a platform is a reliable proxy for an absent *probe* because the *probe* sequence hybridization affinities would be very similar for a cell-isolated nucleotide sequence, leading to similar intensities. For each set, the similarity scores, whose domain is given by [0.0, 1.0], were calculated using the alignment tool in the Phylogenetic Analysis Library [29] and stored as a matrix. The sequence similarity between any two *probes* (Affymetrix probe set-matched target sequences or Agilent spotted sequences) is defined by subtracting an evolutionary distance value from 1.0, where the evolutionary distance whose domain is [0.0, 1.0] between the two sequences was taken as the branch length of the maximum likelihood tree containing only the two sequences, under a set model of substitution [30]. Table 2 shows a toy example of *probe* sequence similarity matrix of 3 platforms: *PF*_*1*_, *PF*_*2*_ and *PF*_*3*_. We define an identifier *PF*-*P*-*G* which is composed of a platform ID, *probe* ID and gene ID. Accordingly, *PF*_*1*_ has 3 *probes P*_*1*_, *P*_*2*_ and *P*_*3*_ and their corresponding genes *G*_*1*_, *G*_*1*_ and *G*_*3*_, i.e., *PF*_*1*_-*P*_*1*_-*G*_*1*_, *PF*_*1*_-*P*_*2*_-*G*_*1*_ and *PF*_*1*_-*P*_*3*_-*G*_*3*_. *PF*_*2*_ has *PF*_*2*_-*P*_*1*_-*G*_*1*_ and *PF*_*2*_-*P*_*2*_-*G*_*2*_, and *PF*_*3*_ has *PF*_*3*_-*P*_*1*_-*G*_*2*_ and *PF*_*3*_-*P*_*2*_-*G*_*3*_. Larger scores represent higher similarities of paired *probe* sequences. In this way, the similarities between any paired *probes* across platforms can be successfully quantified. Take *P*_*3*_ in *PF*_*1*_ as an example, the *probe*-matched *G*_*3*_, i.e., *PF*_*1*_*-P*_*3*_*-G*_*3*_, does not appear in *PF*_*2*_ on which *PF*_*2*_-*P*_*1*_-*G*_*1*_ could be used as a substitute for the *G*_*3*_ since they have the most similar sequences relative to *PF*_*2*_-*P*_*2*_-*G*_*2*_ in sequence.

**Table 2:** **Example of** ***probe*** **sequence similarity matrix**

			**PF**_**1**_			**PF**_**2**_		**PF**_**3**_	
			**P**_**1**_	**P**_**2**_	**P**_**3**_	**P**_**1**_	**P**_**2**_	**P**_**1**_	**P**_**2**_
			**G**_**1**_	**G**_**1**_	**G**_**3**_	**G**_**1**_	**G**_**2**_	**G**_**2**_	**G**_**3**_
PF_1_	P_1_	G_1_	1.0	0.9	0.5	0.8	0.3	0.4	0.1
	P_2_	G_1_		1.0	0.4	0.8	0.2	0.3	0.2
	P_3_	G_3_			1.0	0.4	0.1	0.5	0.9
PF_2_	P_1_	G_1_				1.0	0.0	0.5	0.3
	P_2_	G_2_					1.0	0.8	0.4
PF_3_	P_1_	G_2_						1.0	0.5
	P_2_	G_3_							1.0

#### *Identification of c-LM from multi-connected-datasets*

**Figure 2:**
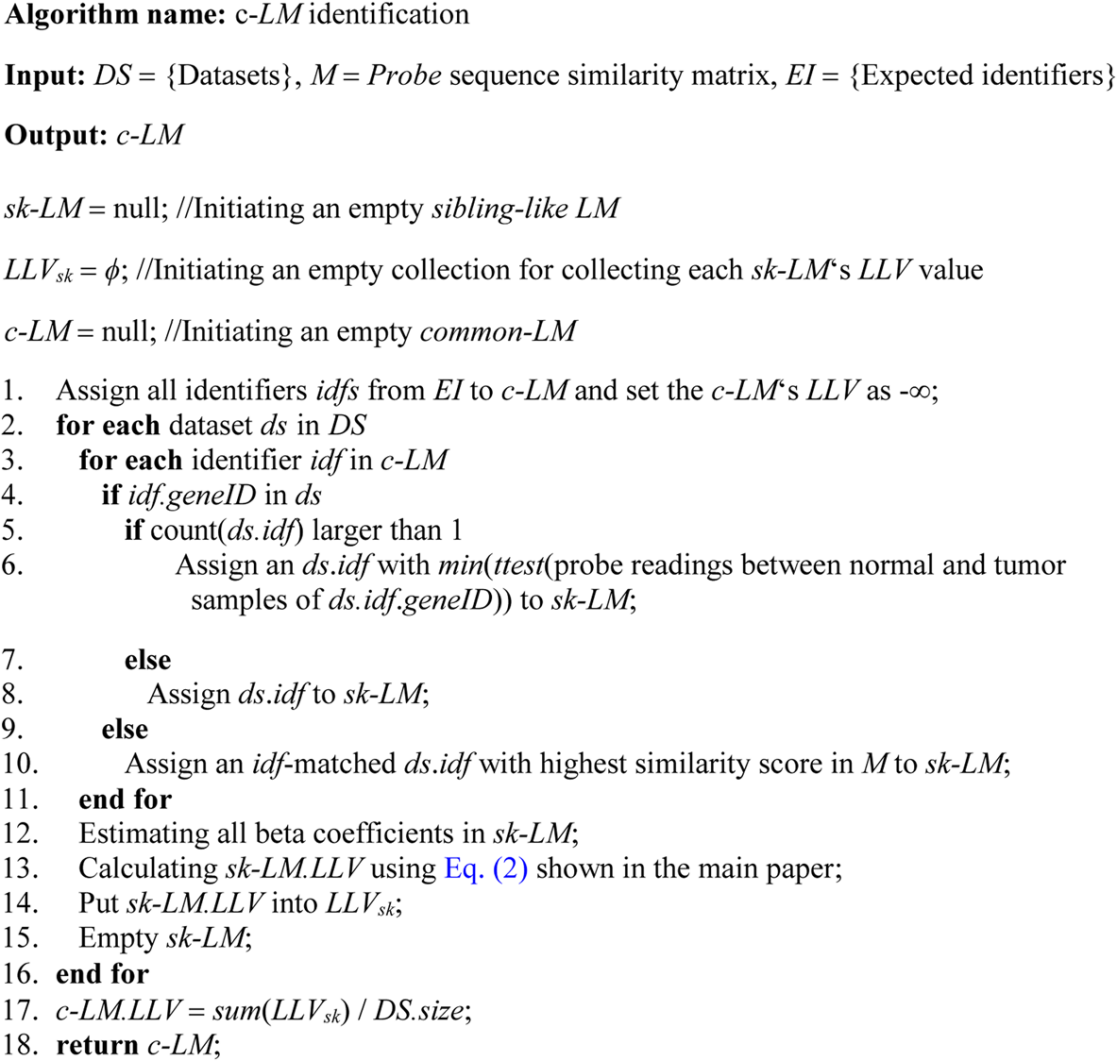
**Algorithm of** ***c-LM*** **identification.**

By referring to the developed *probe* sequence similarity matrix *M*, any *probes* from genes private to a platform can be linked with the most similar substitutes in other platforms. Therefore, all of the platforms in an input set can be connected using the bridge *M*. Figure 2 shows an algorithm to identify a *common logit model* (*c-LM*) from multi-connected datasets. Briefly, the algorithm consists of 5 steps:

1) Input a set *DS* of datasets, a similarity matrix *M*, and a set *EI* of expected/selected identifiers

2) Examine if each dataset contains the gene IDs of the *EI*-contained identifiers

3) For each dataset, by referring to *M*, the substitutes of *EI*-contained identifiers will be assigned to a *sibling-like LM* (*sk-LM*)

4) Calculate each *sk-LM* ‘s *LLV* (natural log likelihood value)

5) Update the *LLV* of the *c-LM* by averaging each dataset-derived *sk-LMs*

Here we extend the toy example stated in the above paragraph to describe this algorithm. Let *DS* = {*DS*_*1*_, *DS*_*2*_, *DS*_*3*_} be three microarray datasets and *M* be the *probe* sequence similarity matrix in Table 2. Assuming *EI* = {*PF*_*1*_*-P*_*1*_*-G*_*1*_, *PF*_*3*_*-P*_*2*_*-G*_*3*_}, a *c-LM* will be introduced to the algorithm *c-LM identification* (Figure 2). The examining identifiers in *sk-LMs* for *DS*_*1*_, *DS*_*2*_ and *DS*_*3*_ will be {*PF*_*1*_*-P*_*1*_*-G*_*1*_, *PF*_*1*_*-P*_*3*_*-G*_*3*_}, {*PF*_*2*_*-P*_*1*_*-G*_*1*_, *PF*_*2*_*-P*_*2*_*-G*_*2*_} and {*PF*_*3*_*-P*_*1*_*-G*_*2*_, *PF*_*3*_*-P*_*2*_*-G*_*3*_} respectively. Then, the *LLV* of the output *c-LM*, with identifiers *PF*_*1*_*-P*_*1*_*-G*_*1*_ and *PF*_*3*_*-P*_*2*_*-G*_*3*_, can be calculated by averaging each *sk-LM*’s *LLV* value. The magnitude of the output the *c-LM*’s *LLV* is based on what identifiers have been defined in *EI*. According to the computational design of a *LM*, the probability of clinical outcomes is attributed to a combined effect of those *probe* intensity values. The next important issue is to properly select these identifiers for including in a model.

### Improving *c-LM* via a heuristic selection process

The *ABs* are defined as a small number of genes *k* with high classification accuracy under the *LM.* Generally, array platforms have tens of thousands of *probes*. The $C_{k}^{n}$ (*n* available *probes* choose *k*) possible *probe* combination of size *k*, and as a result, the cost of determining the *k* genes with the highest classification accuracy will be very high if solving by brute force. For example, if *n* and *k* are 30,000 and 8 respectively, there are approximately 1.6E31 combinations. If testing a combination takes 2 seconds, the total running time of the algorithm will be about 1E24 years, which is unacceptable.

**Figure 3:**
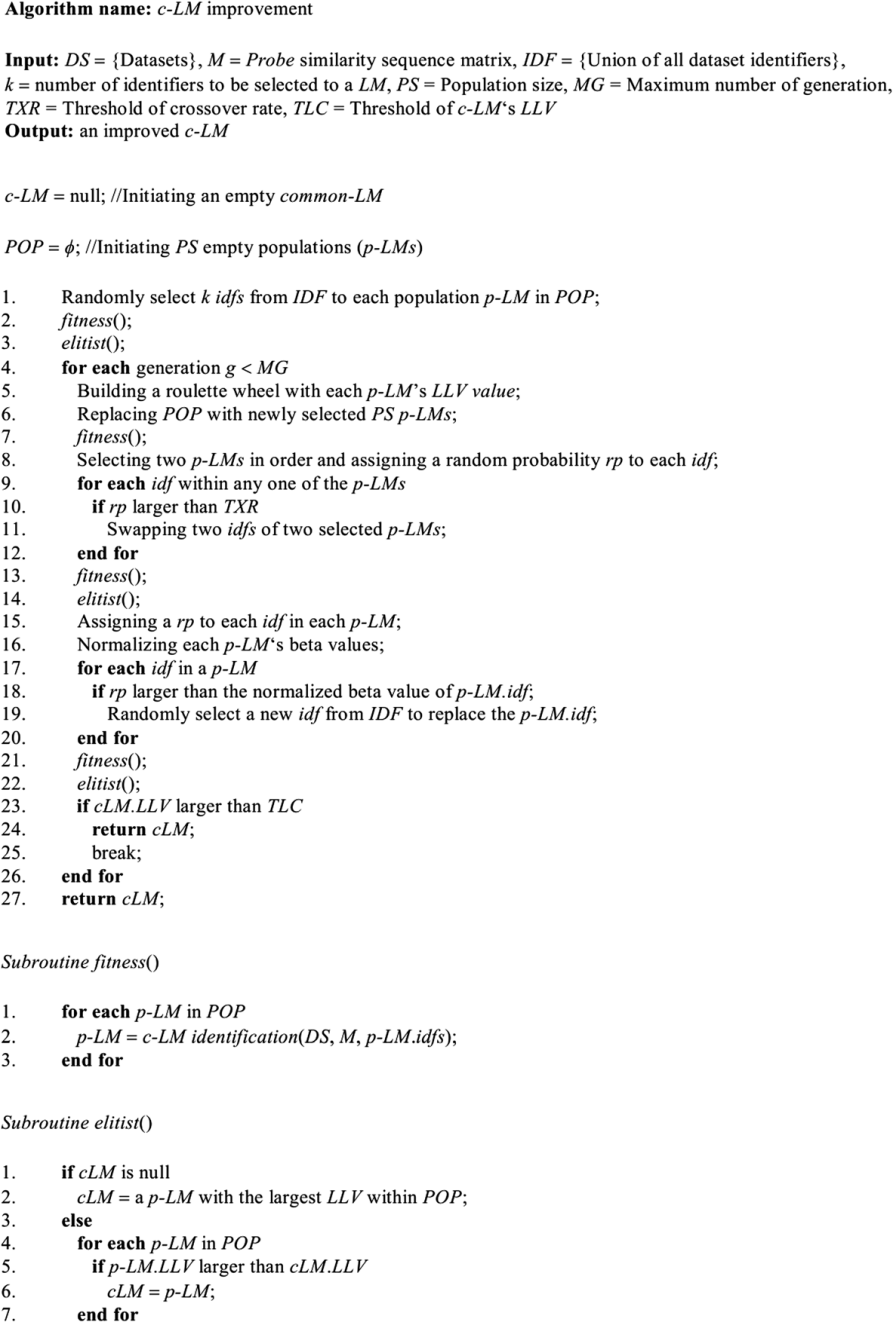
**A genetic algorithm selection for** ***c-LM*** **improvement.**

A genetic algorithm is a heuristic-based approach that was originally designed to efficiently find optimal solutions for a specific fitness function such as the maximum/minimum of a function based on natural evolution including selection, inheritance, mutation and crossover in an iterative process [31]. In this section, we illustrate a genetic algorithm to heuristically improve the *c-LMs* with selected gene candidates in a reasonable time period. An algorithm describing the entire selection process is shown in Figure 3. The backbone of the algorithm consists of 5 steps:

1) Input a set *DS* of datasets, a similarity matrix *M*, a set *IDF* of identifiers derived from the *DS*, a number of identifiers *k* to be selected for each *LM*, a number *PS* of populations in a generation, a maximum number *MG* of generations, a threshold *TXR* of crossover rate, and a threshold *TLC* of the final improved *c-LM*’s *LLV*

2) Select *k* identifiers for each population (*p-LM*) and evaluate the *p-LMs* using the *fitness* subroutine

3) *p-LMs* with larger *LLVs* have higher probabilities to be kept in next generations based on a roulette wheel selection

4) The matched identifiers among the kept populations are swapped with each other or replaced with newly selected identifiers because they were not associated with (small absolute beta values) clinical outcomes

5) Return the final improved *c-LM* derived from the *elitist* subroutine

Based on our empirical tests, we set parameters *PS* = 300, *MG* = 50, *TXR* = 0.5 and *TLC* = 0 (equal to no thresholds) as default setting performed in this study.

### Evaluating improved *c-LM* using a reciprocal test

For evaluating an improved *c-LM* derived from the heuristic selection process, we perform a reciprocal test to examine the model in sample classification accuracy. For each input ESCC/HCC set of datasets, one of the datasets is regarded as a testing dataset and the others are training datasets. Once an improved *c-LM* is successfully trained from the training datasets, the model will then be tested on the testing dataset using leave-one-out cross-validation. The entire evaluation processes are performed using the *KNIME* data mining tool [32]. A formula for calculating the accuracy is given as follows.

$$\mathrm{Accuracy}=\frac{\mathrm{TP}+\mathrm{TN}}{\mathrm{TP}+\mathrm{TN}+\mathrm{FP}+\mathrm{FN}}$$

Where *TP*, *TN*, *FP* and *FN* represent the numbers of true positive, true negative, false positive and false negative, respectively.

## Results and discussion

### Discovering improved *c-LMs* using a genetic algorithm

**Figure 4:**
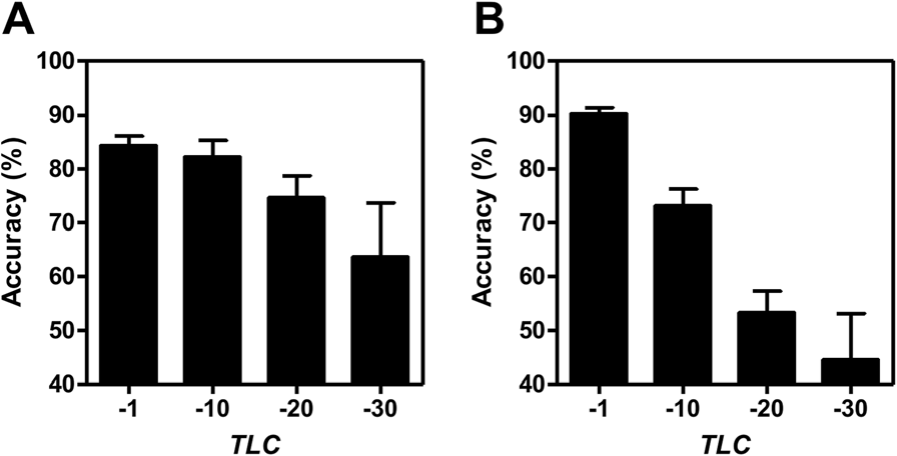
**Examination of improved*c-LMs*using different TLCs. A)***c-LMs* derived from input ESCC set. **B)***c-LMs* derived from input HCC set. Error bars indicate standard error of the means. *TLC*: threshold of *LLVs*.

We tested whether the heuristic genetic algorithm outlined in the previous section is a reliable method for discovering improved *c-LMs* by building *c-LMs* for the ESCC and HCC cancer datasets. In the ESCC input set, an improved *c-LM* can be trained from 3 randomly selected datasets *DS*_*1–2*_, *DS*_*1–3*_ and *DS*_*1–4*_ and tested on the remaining dataset *DS*_*1–1*_. For the HCC dataset, an improved *c-LM* can also be trained from randomly selected datasets *DS*_*2–1*_ and *DS*_*2–3*_ and tested on the remaining dataset *DS*_*2–2*_. For each combination, we repeated the above processes to yield 5 improved *c-LMs* whose *LLV* values exceeded the *TLC* (Threshold of *c-LM’s LLV*) setting. Figure 4 shows the accuracy as a function of different thresholds. We set *TLC* as -1, -10, -20 and -30 (“-30” represents that the final *c-LMs* will not be improved) and set *k* as 3 to observe the changes in sample classification accuracy. Both input sets show the trend that the accuracies increase when the models possess larger *LLVs*. This phenomenon also supports the assertion that the *LLV* is an efficient metric for examining a *LM* given by Eq. (2).

Based on our design for identifying substitute *probe* sequences across platforms, it is possible that we may define incorrect substitutes especially for homologous genes. In this case however, the corresponding *probe* intensities will likely be inconsistent (causing small *LLVs*) and will be eliminated through competition during the selection process of the genetic algorithm. Therefore, the genetic algorithm is reliable for mining improved *c-LMs* with *associated biomarkers* (*ABs*) from the multi-connected datasets.

### Considering more datasets yields better accuracy compared to increasing the gene number

**Figure 5:**
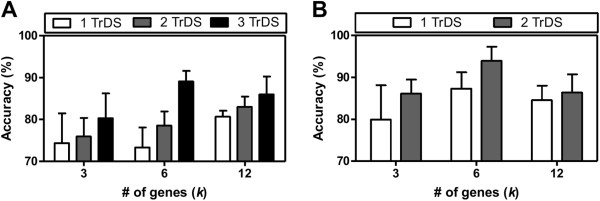
**Testing improved*c-LMs*built by different number of training datasets. A)** Accuracy as a function of various ESCC training datasets, grouped by gene numbers. **B)** Accuracy as a function of various HCC training datasets, grouped by gene numbers. Error bars indicate standard error of the means. TrDS: number of training datasets.

**Figure 6:**
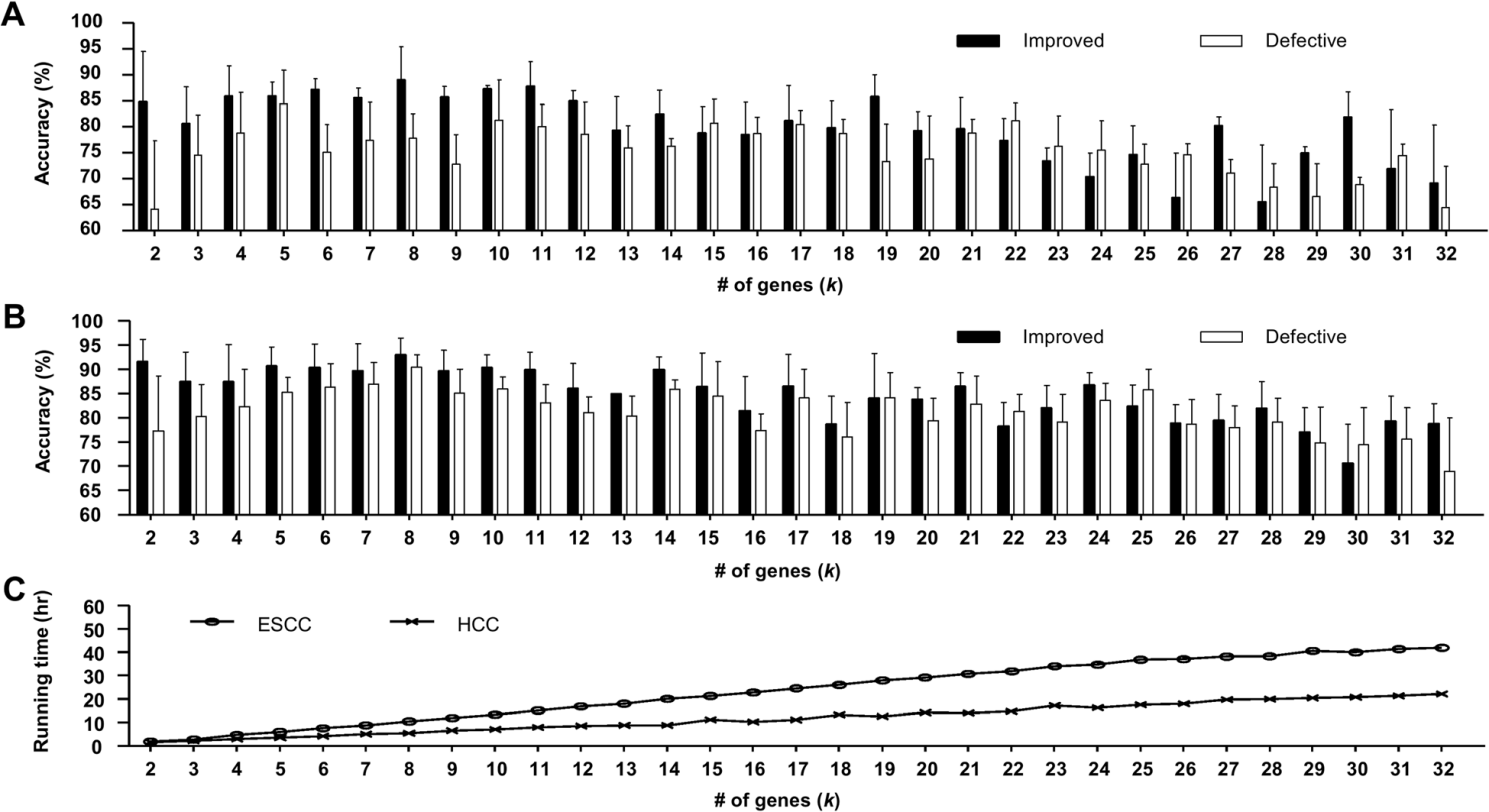
**Scalability of improved*c-LMs.*A)** Accuracy as a function of various ESCC training datasets, grouped by gene numbers. **B)** Accuracy as a function of various HCC training datasets, grouped by gene numbers. **C)** Average running times of panel **A** and **B**. Error bars indicate standard error of the means. Improved: improved *c-LMs*; Defective: removing one out of an improved *c-LM*-contained genes by turns.

As in other previously published meta-analysis approaches, the two most important factors affecting our method are the number of data sources and predictor variables/genes. Based on a reciprocal test, we examined the scalability of our improved *c-LMs* in the number of independent datasets to be used as training datasets and in the number of genes to be selected in a model, i.e., the parameter *k*. Figure 5A and B show the accuracy as a function of various combinations of training datasets from the ESCC and HCC datasets respectively. The average accuracies of improved *c-LMs* derived from more training sets were higher than those derived from fewer training datasets when setting the *AB* number *k* as 3, 6 and 12. Therefore, based on the same reciprocal test, we developed the improved *c-LMs* from 3 ESCC and 2 HCC training datasets for addressing the other issue: how many predictor variables/genes are suitable for a model in cancer sample classification. We adjusted *k* from 2 to 32 and calculated the accuracy in both input sets as shown in Figure 6A and B. The average accuracy 84.2% for smaller *k* (*k* = 2 ~ 16) decreased to 75.7% (*k* = 17 ~ 32) in the ESCC input set. A similar trend was also observed in the HCC set (88.6% to 80.9%), likely due to overfitting. The decrease in accuracy for higher values of *k* shows that considering more variables/genes in a model does not always improve classification performance. Coincidently, the improved *c-LMs* in the both sets have the highest accuracies when *k* was set as 8. This number would be an ideal default setting for experimentalists who do not have a hypothesis regarding the number of genes of interest in advance. Another merit is that using fewer biomarkers requires less computational resources and is easier to validate and follow up on for further biological insight.

### Improved *c-LMs* reveal *ABs*

Based on the fundamental principle of the model design, the target class (tumor/normal) should be described by a combination effect of several independent variables. Therefore, the genes in the improved *c-LMs* should be strongly associated with each other in the biological event of interest. To verify this, we compared our improved *c-LMs* and their corresponding “defective” *c-LMs* by removing one of the *associated biomarkers* (*ABs*) by turns in each *c-LM*, i.e. remove a different *AB k* times and retest the models with *k*-1 *ABs*, for the two input sets as shown in Figure 6. The accuracies of the improved *c-LMs* are on average higher (4.5% in ESCC and 3.4% in HCC) than the corresponding defective models. Furthermore, as stated in the previous section, the models seem to be overfitting for increasing values of *k*. The average deviations in accuracy between the improved and defective *c-LMs* decreases from 7.1% (*k* = 2 ~ 16) to 2.0% (*k* = 17 ~ 32) in ESCC (Figure 6A) and from 5.1% (*k* = 2 ~ 16) to 1.8% (*k* = 17 ~ 32) in HCC (Figure 6B). Therefore, removing *ABs* from models that are not overfitting causes a larger decline in accuracy relative to the overfitting models due to the combination effect of the *ABs*.

We also measured the running times for the scalability experiments (Figure 6A and B) and display the results in Figure 6C. The running time for both input sets scales linearly as we increase *k*; this linear trend would be significantly better than a brute force manner (data not shown).

### *ABs* are highly related to cancer development and connected in network

In addition to using the reciprocal tests (models were trained from training datasets and then tested on remaining testing datasets) to repetitively examine the improved *c-LMs* in different cancer-related datasets, we additionally tested whether the *associated biomarkers* (*ABs*) we discovered possessed biological insights into cancer development using a GO (Gene Ontology) enrichment analysis [33]. We also evaluated the location of *ABs* in a biological network derived from diverse data types such as protein-protein, gene regulatory, DNA-protein, and RNA-protein interactions, since some previous studies have indicated that mining associated gene-based patterns like association rules [4], co-expressed patterns [34] and sequential patterns [35,36] from single experiments are related in biological networks. Therefore, we chose to examine the distance between *ABs* in the IPA (Ingenuity® Systems, http://www.ingenuity.com) interaction network.

**Figure 7:**
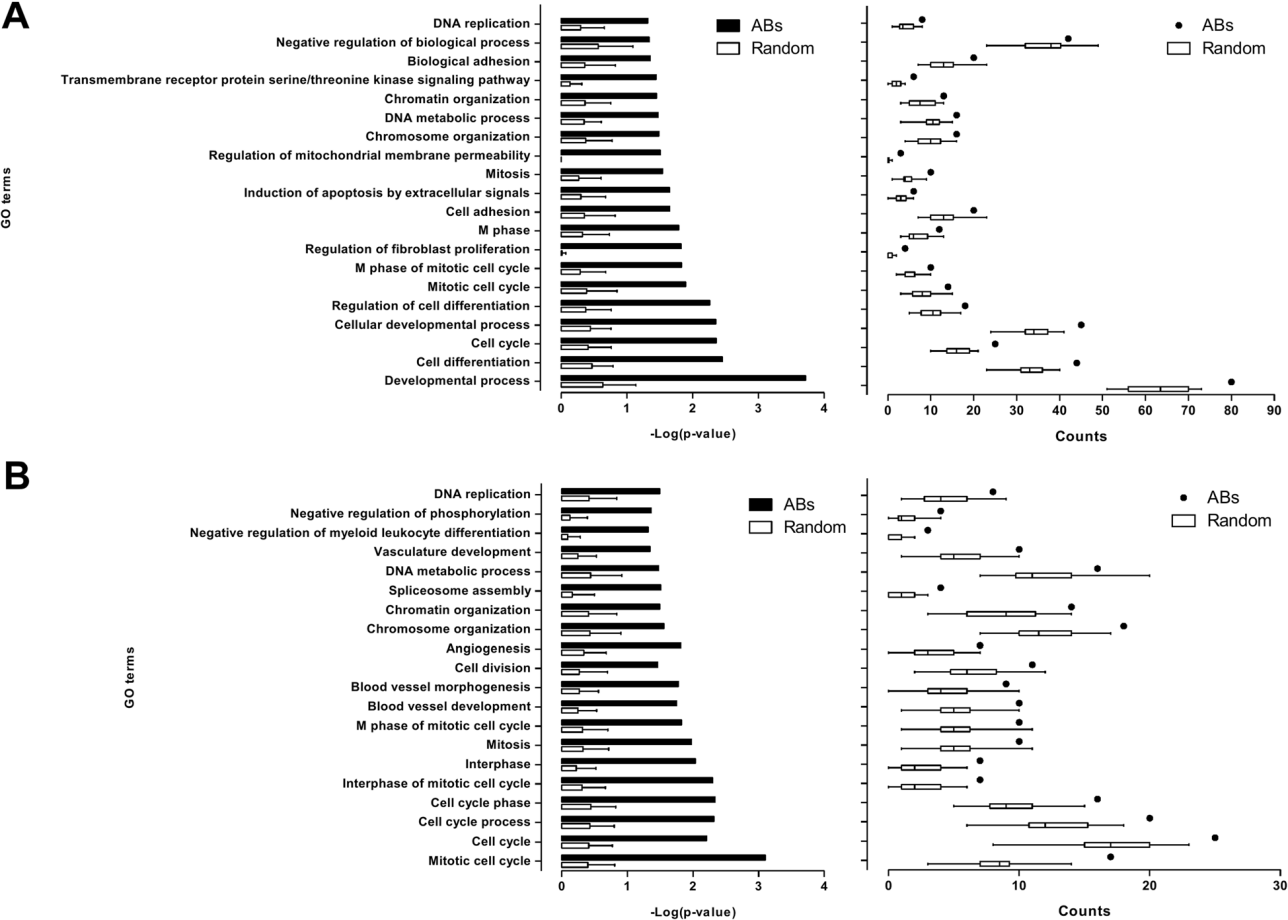
**Enrichment analysis of cancer-related GO terms. A)** Performing the test in ESCC set. **B)** Performing the test in HCC set. *ABs*: associated biomarkers; Random: randomly selected genes from array platforms. Error bars indicate standard error of the means.

**Figure 8:**
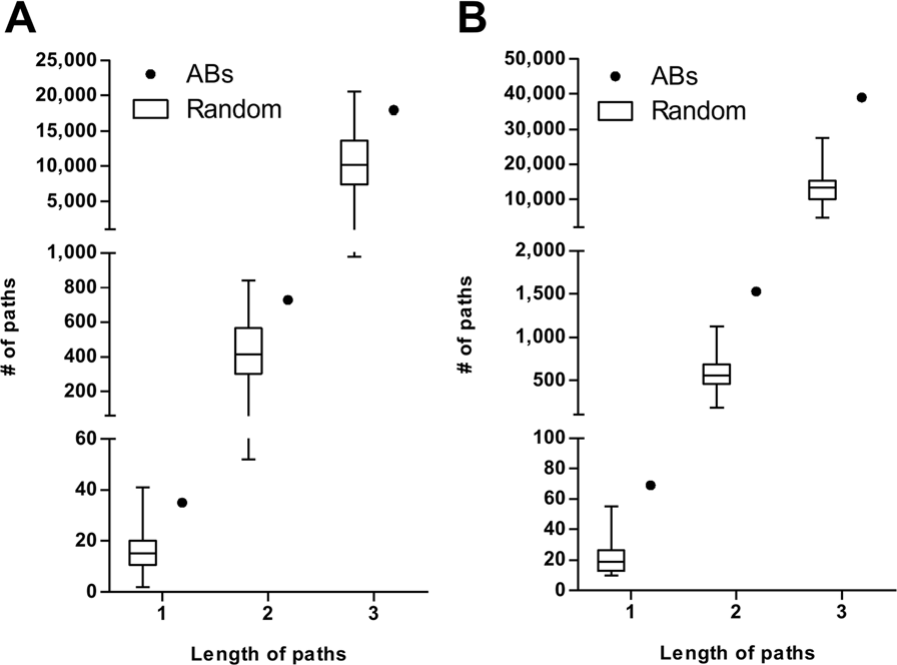
**Number of shortest paths among genes in network. A)** Number of shortest paths as a function of different lengths for ESCC input set. **B)** Number of shortest paths as a function of different lengths for HCC input set. *ABs*: associated biomarkers; Random: randomly selected genes from array platforms.

**Figure 9:**
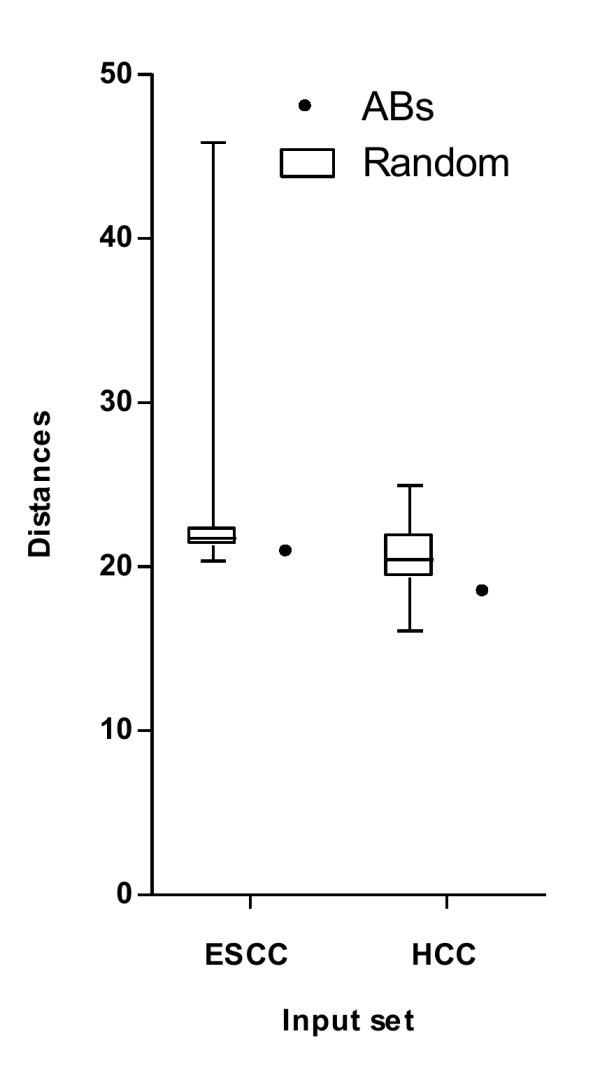
**Distance of shortest paths among genes in network.** The distance of shortest paths among genes for ESCC and HCC input sets. *ABs*: associated biomarkers; Random: randomly selected genes from array platforms.

As noted in Figure 6A and B, the improved *c-LMs* for both input sets have the highest average accuracies when *k* is set as 8. We therefore set *k* as 8 and repeatedly executed *MiningABs* to build 48 improved *c-LMs* from each input set, i.e., 48 improved *c-LMs* (Additional file 1: Table S1) were trained from 4 ESCC datasets: DS_1–1_, DS_1–2_, DS_1–3_ and DS_1–4_, and 48 improved *c-LMs* (Additional file 1: Table S2) were trained from 3 HCC datasets: DS_2–1_, DS_2–2_ and DS_2–3_. For each set, 305 (ESCC) and 288 (HCC) out of 384 (48 × 8) *ABs* were distinct. To evaluate whether the two sets of distinct *ABs* have a high propensity for cancer development, they were analyzed separately using a GO enrichment analysis with significant p-values smaller than 0.05 through the DAVID tool [33]. All of the cancer-related GO terms among the resulting outputs are shown in Figure 7, and their corresponding genes are listed in Additional file 2: Table S3 and S4. For achieving a fair comparison, we randomly selected the same number of genes 30 times and repeatedly performed the same testing process as a control group. Overall, the distinct *ABs* were more highly enriched in cancer-related GO terms compared to the randomly selected genes in both input sets (left panel in Figure 7A and B). Furthermore, within each GO term, the number of distinct *ABs* was also on average higher than that of the randomly selected genes (right panel in Figure 7A and B). We also manually uploaded the distinct *ABs* and the randomly selected genes to IPA to determine the number of shortest paths of zero (path length = 1), one (path length = 2) and two (path length = 3) intermediates among the individually uploaded gene sets. Here we provide a toy example to illustrate the definition of different path lengths in Additional file 3: Figure S1. The number of shortest paths was higher for our distinct *ABs* compared to the randomly selected genes in both input sets (Figure 8). A similar result in terms of the distance of shortest paths among genes could also be seen in another publicly available database called HGC [37]. Overall, the average distances among *ABs* in the gene connectome network were shorter/closer than the average distances among the randomly selected genes (Figure 9). Therefore, this evaluation demonstrates that the improved *c-LMs*-involved *ABs* are strongly associated with cancer development as well as highly connected in a biological network.

Among the distinct *ABs*, 10.8% (33/305 in ESCC) and 16.3% (47/288 in HCC) of the genes were included in at least two models. These overlapped genes ordered by their number of appearances across models are shown in Table 3. In terms of frequency of occurrence, the top three ranked candidate genes in the ESCC set were *COL3A1* (collagen, type III, alpha 1), *COL1A2* (collagen, type I, alpha 2) and *FNDC3B* (fibronectin type III domain containing 3B), and in the HCC set were *PPIA* (Peptidylprolyl Isomerase A (Cyclophilin A)), *CXCL14* (chemokine (C-X-C motif) ligand 14), *CAP2* (CAP, adenylate cyclase-associated protein, 2 (yeast)), *CDKN2A* (cyclin-dependent kinase inhibitor 2A) and *ECM1* (extracellular matrix protein 1). Coincidently, the most-observed (top-1) gene *COL3A1* and *COL1A2* (top-2) for ESCC, seen across at least 9 models, were also identified by Su *et al.* in the original input dataset [5]. *COL3A1* was consistently expressed in people with a family history of upper gastrointestinal cancer [38]. This gene has been identified as a potential biomarker in human cutaneous squamous cell carcinoma tissue samples and cell lines [39]. Additionally, up-regulation of *COL1A2* expression has been identified to be significantly associated with early ESCC in another Chinese population [40]. Although *FNDC3B* has not been reported as biomarker, it co-occurred with the top-1 *COL3A1* in two improved *c-LMs* (#13 and #30 in Additional file 1: Table S1).

**Table 3:** **List of ABs observed in multiple improved** ***c-LMs***

**Input set**	**Overlapped** ***ABs*** **(N)**
ESCC	COL3A1(10), COL1A2(9), FNDC3B(8), ID4(7), PMEPA1(7), BID(6), CDH11(4), COL11A1(4), ETV5(4), AGRN*(3), DHRS7(3), MMP11(3), TOM1L2(3), VPS8(3), ASXL1(2), C9orf100*(2), CHEK1(2), ECT2(2), FST(2), HMGB3(2), IGF2BP2(2), KIF14(2), KIRREL(2), NETO2(2), PGM5(2), PKD1*(2), PSME4(2), RPS20*(2), RUNX1*(2), STYXL1(2), TCFL5(2), TOB1(2), TSGA14(2)
HCC	PPIA(14), CXCL14(8), CAP2(6), CDKN2A(6), ECM1(6), APBA2BP(5), CLEC4M(5), ACLY(4), CXCL12(4), CYP1A2(4), SAC3D1(4), COX8A(3), FOS(3), SHFM1(3), SNRPE(3), SUB1(3), YWHAQ(3), AKR1C3(2), ATP6V0E1(2), BMI1(2)), CELSR3*(2), CUL4B(2), DR1(2), FASTK(2), FHIT(2), FLAD1(2), GABRP(2), GM2A(2), GTPBP9(2), HBB(2), ITGA6(2), LOC390998*(2), MEA1(2), MRPS35(2), NPM1(2), PCDHGC3*(2), PDCD5(2), PPP2R5A(2), PRKDC(2), REEP6*(2), SNW1(2), STAB2(2), TMED9(2), TNS1(2), VAMP4(2), XLKD1(2), ZRF1(2)

ESCC: esophageal squamous cell carcinoma; HCC: hepatocellular carcinoma; N: # of appearance times; *: uncommon genes across a set of array platforms.

We postulated that a classifier based on gene expression of the genes from the discovered models was more likely to be applicable with a high confidence in the clinic. For the HCC set, the most-observed (top-1) gene *PPIA* has been characterized as a biomarker for the diagnosis of liver cancer under a patent Publication Number “US20100203510 A1”. Sun *et al.* reported that protein CXCL14 (top-2) is a member of 8 markers complementary to a currently used marker, alpha-fetoprotein (AFP) [41]. Interestingly, the *CXCL14* gene also co-occurred with *PPIA* in three of our improved *c-LMs* (#11, #14 and #22 in Additional file 1: Table S2). We postulated that this gene might be the best proxy for the AFP. For the rest of the top three ranked genes including *CAP2*, *CDKN2A* and *ECM1*, Sakamoto *et al.* reported that both *CAP2* and *HSP70* (heat shock protein 70) were molecular markers for early HCC detection [42], *CDKN2A* has been shown as a diagnostic and prognostic molecular marker through its epigenetic alteration in HCC [43], and *ECM1* was identified as a prognostic factor associated with metastatic potential of HCC [44]. It is well known that cancer development is not caused by a group of unassociated genes. For this reason, we took into account the associations among these individual genes in this study. By comparing the results for the top three ranked candidate *ABs* in both input sets to previously published results, we find that the genes in the candidate *ABs* are biologically relevant. Additionally, the genes associated with the top-1 genes *COL3A1* (ESCC) and *PPIA* (HCC) may provide important biological information that can help identify the function of these genes in their respective cancers.

Among the *ABs* of *c-LMs* shown in Additional file 1: Table S1 and S2, we can observe that certain genes are not common to all microarray platforms in the input datasets yet and are capable of being associated with other genes on the platforms to achieve accurate cancer classification. If we only focused on the common genes, these valuable genes might be lost from the resulting *ABs*.

Therefore, mining *ABs* using genes not common to the microarray platforms allows for new potentially relevant genes to be discovered. These findings may be very important to biologists for investigating putative cancer mechanisms and identifying drug targets.

## Conclusions

In this study, we developed an approach to efficiently identify *associated biomarkers* (*ABs*) across different array-based platforms. We then successfully developed a new meta-analysis method called *MiningABs* for mining *ABs* using an improved *common logit-model* (*c-LM*). Finally, we evaluated our method using 2 cancer (esophageal squamous cell carcinoma and hepatocellular carcinoma) gene expression datasets as a case study to demonstrate the utility of *MiningABs* for cancer biology. The main results of *MiningABs* include: i) by measuring the similarities among any paired *probe* sequences to link different platforms, the resulting *ABs* are not limited to the genes common to all platforms, ii) in our scalability experiment, we demonstrated that any one gene in a group of *ABs* was necessarily required for high cancer sample classification accuracy, iii) in terms of efficiency, the running time of the *MiningABs* does not increase exponentially when mining for larger sets of *ABs*, and iv) testing our *ABs* using GO enrichment, a literature survey, and a network analysis indicated that our *ABs* are not only strongly associated with cancer development but also highly connected in a biological network, supporting the biological validity of the *ABs*.

There are several extensions that can be performed. According to our computational design, a *c-LM* is developed by combining a few *sibling-like LMs* that were derived from each dataset. Over tens of thousands of iteratively-derived *c-LMs* are evaluated and improved using the genetic algorithm. These processes could be executed in a parallel way on a GPU (graphics processing unit) to reduce the time cost.

## Abbreviations

MiningABs: Mining associated biomarkers; ABs: Associated biomarkers; LM: Logit model; sk-LM: sibling-like logit model; c-LM: common logit model; ESCC: Esophageal squamous cell carcinoma; HCC: Hepatocellular carcinoma; GEO: Gene Expression Omnibus; LLV: Natural log likelihood value; *TLC*: Threshold of c-LM’s LLV.

## Competing interests

The authors declare that they have no competing interests.

## Authors’ contributions

CPC and CD wrote the paper. CPC and YCL developed the software and conducted the original experiments. CPC, CD and YCL conceived and designed the experiments. CPC and YCL analyzed the experimental results. KAF and VST supervised the study. CPC, CD, KAF, YCL and VST read and approved the final manuscript.

## Supplementary Material

Additional file 1 **List of 48 improvedc-*LMs*** **(*k* = 8) trained from all datasets in ESCC and HCC input sets.** Click here for file

Additional file 2 **List of** ***ABs*** **involved in cancer-related GO terms for the ESCC and HCC input sets.** Click here for file

Additional file 3 Example of different path lengths examined in this study. Click here for file
