# Supplementary material for: MiningABs: mining associated biomarkers across multi-connected gene expression datasets
Source: BMC Bioinformatics. 2014 Jun 8;15:173. doi: 10.1186/1471-2105-15-173 (PMC4068973; doi:10.1186/1471-2105-15-173)
Supplement: Additional file 1 — List of 48 improvedc- LMs ( k = 8) trained from all datasets in ESCC and HCC input sets. [file 1471-2105-15-173-S1.doc]

**Table S1.** List of 48 improved c-*LMs* (*k*=8) trained from all datasets in ESCC input set.

| # | 1^st^ *AB* | 2^nd^ *AB* | 3^rd^ *AB* | 4^th^ *AB* | 5^th^ *AB* | 6^th^ *AB* | 7^th^ *AB* | 8^th^ *AB* | *LLV* |
| --- | --- | --- | --- | --- | --- | --- | --- | --- | --- |
| 1 | GSE23400(GPL96):211466_at:4781:NFIB | GSE23400(GPL96):220858_at:8470:SORBS2 | GSE20347:205170_at:6773:STAT2 | GSE20347:214674_at:10869:USP19 | GSE23400(GPL97):225032_at:64778:FNDC3B | GSE23400(GPL96):216231_s_at:567:B2M | GSE20347:216247_at:6224:RPS20* | GSE23400(GPL96):204751_x_at:1824:DSC2 | -2.37E-11 |
| 2 | GSE20347:221736_at:57148:KIAA1219 | GSE29001:218089_at:25980:C20orf4* | GSE20347:218888_s_at:81831:NETO2 | GSE23400(GPL96):212234_at:171023:ASXL1 | GSE23400(GPL96):202079_s_at:22906:TRAK1 | GSE20347:202724_s_at:2308:FOXO1 | GSE23400(GPL97):235185_s_at:388692:LOC388692* | GSE29001:215076_s_at:1281:COL3A1 | -3.63E-11 |
| 3 | GSE23400(GPL96):204948_s_at:10468:FST | GSE20347:220574_at:80031:SEMA6D | GSE29001:204521_at:29902:C12orf24* | GSE20347:218321_x_at:51657:STYXL1 | GSE23400(GPL96):207164_s_at:10472:ZNF238* | GSE20347:217875_s_at:56937:PMEPA1 | GSE29001:201709_s_at:8508:NIPSNAP1* | GSE20347:209221_s_at:9885:OSBPL2* | -5.62E-11 |
| 4 | GSE29001:204320_at:1301:COL11A1 | GSE29001:215637_at:95681:TSGA14 | GSE23400(GPL97):223268_at:28970:C11orf54* | GSE20347:201188_s_at:3710:ITPR3 | GSE23400(GPL96):222333_at:259173:ALS2CL | GSE23400(GPL96):220825_s_at:55243:KIRREL | GSE20347:202663_at:7456:WIPF1 | GSE20347:219611_s_at:64793:CCDC21 | -1.59E-10 |
| 5 | GSE29001:216113_at:10152:ABI2 | GSE20347:202339_at:8189:SYMPK* | GSE29001:209903_s_at:545:ATR | GSE20347:202403_s_at:1278:COL1A2 | GSE29001:211725_s_at:637:BID | GSE23400(GPL97):223963_s_at:10644:IGF2BP2 | GSE29001:218878_s_at:23411:SIRT1* | GSE23400(GPL96):215019_x_at:84436:ZNF528* | -3.15E-11 |
| 6 | GSE29001:208543_at:26538:OR10H2* | GSE23400(GPL97):232564_at:6553:SLC9A5 | GSE23400(GPL97):226828_s_at:26508:HEYL | GSE23400(GPL97):222449_at:56937:PMEPA1 | GSE29001:208491_s_at:5239:PGM5 | GSE23400(GPL97):227143_s_at:637:BID | GSE23400(GPL96):216875_x_at:55547:HAB1* | GSE23400(GPL97):236179_at:1009:CDH11 | -2.13E-11 |
| 7 | GSE23400(GPL97):235781_at:774:CACNA1B | GSE29001:207902_at:3568:IL5RA* | GSE23400(GPL96):201852_x_at:1281:COL3A1 | GSE20347:202295_s_at:1512:CTSH* | GSE29001:221436_s_at:83461:CDCA3 | GSE23400(GPL97):236937_at:23355:VPS8 | GSE29001:207173_x_at:1009:CDH11 | GSE23400(GPL96):217419_x_at:375790:AGRN* | -2.89E-11 |
| 8 | GSE23400(GPL97):235239_at:169714:QSOX2* | GSE23400(GPL97):229218_at:1278:COL1A2 | GSE29001:219560_at:79680:C22orf29 | GSE20347:212986_s_at:11011:TLK2 | GSE20347:221401_at:27091:CACNG5* | GSE23400(GPL96):219787_s_at:1894:ECT2 | GSE23400(GPL96):206074_s_at:3159:HMGA1* | GSE23400(GPL96):211630_s_at:2937:GSS* | -4.61E-11 |
| 9 | GSE29001:203106_s_at:27072:VPS41 | GSE29001:204223_at:5549:PRELP | GSE23400(GPL97):222449_at:56937:PMEPA1 | GSE20347:210785_s_at:9473:C1orf38* | GSE23400(GPL96):204778_x_at:3217:HOXB7* | GSE23400(GPL96):213008_at:55215:FANCI | GSE29001:52169_at:92335:STRADA | GSE23400(GPL97):226666_at:23002:DAAM1 | -4.25E-11 |
| 10 | GSE20347:207982_at:3010:HIST1H1T* | GSE20347:203970_s_at:8504:PEX3* | GSE20347:202328_s_at:5310:PKD1* | GSE23400(GPL96):201852_x_at:1281:COL3A1 | GSE23400(GPL97):225442_at:4921:DDR2 | GSE29001:216999_at:2057:EPOR* | GSE29001:212175_s_at:204:AK2* | GSE29001:212302_at:23168:RTF1 | -4.20E-11 |
| 11 | GSE29001:207797_s_at:55805:LRP2BP* | GSE23400(GPL96):212708_at:339287:MSL1 | GSE20347:212619_at:23306:TMEM194A* | GSE29001:208129_x_at:861:RUNX1* | GSE23400(GPL97):225697_at:51755:CRKRS | GSE23400(GPL97):241394_at:284120:LOC284120* | GSE23400(GPL97):227750_at:8997:KALRN | GSE23400(GPL97):229271_x_at:1301:COL11A1 | -6.99E-11 |
| 12 | GSE23400(GPL97):229218_at:1278:COL1A2 | GSE23400(GPL97):235266_at:29028:ATAD2 | GSE20347:201789_at:51635:DHRS7 | GSE29001:221866_at:7942:TFEB* | GSE23400(GPL96):219374_s_at:79796:ALG9 | GSE23400(GPL97):227088_at:8654:PDE5A | GSE23400(GPL96):206614_at:8200:GDF5* | GSE29001:208491_s_at:5239:PGM5 | -2.14E-11 |
| 13 | GSE20347:218618_s_at:64778:FNDC3B | GSE20347:203976_s_at:10036:CHAF1A | GSE23400(GPL96):201852_x_at:1281:COL3A1 | GSE20347:216855_s_at:3192:HNRNPU | GSE29001:214115_at:10791:VAMP5* | GSE23400(GPL96):209614_at:125:ADH1B | GSE23400(GPL96):216640_s_at:10130:PDIA6* | GSE20347:205393_s_at:1111:CHEK1 | -4.93E-11 |
| 14 | GSE29001:206208_at:762:CA4* | GSE23400(GPL96):203876_s_at:4320:MMP11 | GSE29001:219634_at:50515:CHST11 | GSE23400(GPL96):202265_at:648:BMI1* | GSE23400(GPL96):209553_at:23355:VPS8 | GSE20347:209947_at:9898:UBAP2L* | GSE23400(GPL96):220092_s_at:84168:ANTXR1 | GSE29001:221803_s_at:29982:NRBF2 | -2.65E-08 |
| 15 | GSE23400(GPL96):212219_at:23198:PSME4 | GSE20347:218745_x_at:54929:TMEM161A* | GSE23400(GPL97):233743_x_at:53637:S1PR5 | GSE29001:218041_x_at:54407:SLC38A2 | GSE29001:218149_s_at:55893:ZNF395 | GSE20347:221906_at:114112:TXNRD3* | GSE29001:201852_x_at:1281:COL3A1 | GSE23400(GPL97):224947_at:79102:RNF26* | -3.93E-11 |
| 16 | GSE29001:218693_at:23555:TSPAN15* | GSE23400(GPL96):213397_x_at:6038:RNASE4* | GSE29001:209662_at:1070:CETN3* | GSE29001:34449_at:835:CASP2 | GSE23400(GPL96):203311_s_at:382:ARF6 | GSE29001:56829_at:83696:TRAPPC9* | GSE29001:210788_s_at:51635:DHRS7 | GSE29001:201852_x_at:1281:COL3A1 | -4.66E-11 |
| 17 | GSE23400(GPL97):226198_at:146691:TOM1L2 | GSE20347:204709_s_at:9493:KIF23 | GSE23400(GPL97):224139_at:220070:C11orf76* | GSE23400(GPL96):222191_s_at:11285:B4GALT7* | GSE23400(GPL96):204242_s_at:8310:ACOX3* | GSE20347:207345_at:10468:FST | GSE23400(GPL96):219559_at:63910:C20orf59 | GSE20347:35846_at:7067:THRA* | -6.10E-11 |
| 18 | GSE20347:64440_at:84818:IL17RC | GSE23400(GPL97):222449_at:56937:PMEPA1 | GSE23400(GPL97):224877_s_at:64969:MRPS5* | GSE20347:204126_s_at:8318:CDC45L* | GSE23400(GPL97):228834_at:10140:TOB1 | GSE23400(GPL97):233517_s_at:64344:HIF3A | GSE29001:207446_at:10333:TLR6* | GSE23400(GPL96):219476_at:79098:C1orf116 | -8.82E-11 |
| 19 | GSE20347:203083_at:7058:THBS2* | GSE29001:211725_s_at:637:BID | GSE23400(GPL96):213750_at:26156:RSL1D1* | GSE23400(GPL97):235296_at:56648:EIF5A2 | GSE29001:213634_s_at:55687:TRMU* | GSE29001:219633_at:79183:TTPAL | GSE20347:201513_at:7247:TSN* | GSE23400(GPL97):224170_s_at:56995:TULP4 | -4.54E-11 |
| 20 | GSE29001:213635_s_at:6294:SAFB* | GSE23400(GPL97):226198_at:146691:TOM1L2 | GSE20347:218294_s_at:10762:NUP50 | GSE23400(GPL97):229218_at:1278:COL1A2 | GSE29001:209070_s_at:8490:RGS5 | GSE20347:202478_at:28951:TRIB2* | GSE20347:200949_x_at:6224:RPS20* | GSE23400(GPL96):205759_s_at:6820:SULT2B1* | -1.04E-05 |
| 21 | GSE23400(GPL97):238601_at:5257:PHKB | GSE23400(GPL96):208760_at:7329:UBE2I | GSE29001:211833_s_at:581:BAX* | GSE23400(GPL96):201852_x_at:1281:COL3A1 | GSE29001:221815_at:11057:ABHD2 | GSE29001:200028_s_at:56910:STARD7* | GSE20347:205662_at:27077:B9D1* | GSE20347:206550_s_at:9631:NUP155* | -1.63E-09 |
| 22 | GSE23400(GPL97):222606_at:55055:ZWILCH | GSE23400(GPL97):228703_at:283208:P4HA3* | GSE23400(GPL96):218888_s_at:81831:NETO2 | GSE20347:220326_s_at:55701:FLJ10357 | GSE23400(GPL96):38892_at:23506:KIAA0240* | GSE29001:219787_s_at:1894:ECT2 | GSE23400(GPL97):231032_at:286071:LOC286071* | GSE23400(GPL96):218928_s_at:54020:SLC37A1 | -6.10E-11 |
| 23 | GSE23400(GPL97):228144_at:91975:ZNF300* | GSE29001:211718_at:84789:MGC2889* | GSE23400(GPL97):222450_at:56937:PMEPA1 | GSE20347:217062_at:1760:DMPK* | GSE29001:209780_at:57157:PHTF2* | GSE23400(GPL97):226933_s_at:3400:ID4 | GSE20347:212234_at:171023:ASXL1 | GSE23400(GPL97):236166_at:285147:LOC285147* | -1.99E-09 |
| 24 | GSE23400(GPL96):207305_s_at:22878:KIAA1012* | GSE23400(GPL96):202403_s_at:1278:COL1A2 | GSE23400(GPL97):224855_at:29920:PYCR2* | GSE23400(GPL97):226028_at:54538:ROBO4 | GSE20347:209754_s_at:7112:TMPO | GSE20347:214751_at:90333:ZNF468* | GSE23400(GPL96):207907_at:8740:TNFSF14* | GSE20347:211142_x_at:3111:HLA-DOA | -2.99E-11 |
| 25 | GSE23400(GPL96):205684_s_at:55667:DENND4C | GSE29001:210805_x_at:861:RUNX1* | GSE29001:220902_at:196707:FLJ12616* | GSE29001:218618_s_at:64778:FNDC3B | GSE29001:218073_s_at:55706:TMEM48 | GSE29001:217301_x_at:5928:RBBP4 | GSE23400(GPL96):215637_at:95681:TSGA14 | GSE20347:219511_s_at:9627:SNCAIP | -2.39E-08 |
| 26 | GSE20347:211481_at:6579:SLCO1A2* | GSE23400(GPL96):202404_s_at:1278:COL1A2 | GSE20347:218847_at:10644:IGF2BP2 | GSE23400(GPL97):230522_s_at:84904:C9orf100* | GSE23400(GPL97):236641_at:9928:KIF14 | GSE23400(GPL97):222533_at:51185:CRBN | GSE29001:212220_at:23198:PSME4 | GSE20347:221453_at:57818:G6PC2* | -2.42E-11 |
| 27 | GSE20347:212591_at:23029:RBM34* | GSE23400(GPL96):202404_s_at:1278:COL1A2 | GSE23400(GPL96):209791_at:11240:PADI2* | GSE29001:203744_at:3149:HMGB3 | GSE23400(GPL97):227103_s_at:9718:ECE2 | GSE23400(GPL96):204605_at:10668:CGRRF1* | GSE23400(GPL96):210788_s_at:51635:DHRS7 | GSE20347:209292_at:3400:ID4 | -1.97E-11 |
| 28 | GSE29001:217279_x_at:4323:MMP14* | GSE29001:218618_s_at:64778:FNDC3B | GSE29001:201630_s_at:52:ACP1* | GSE23400(GPL96):203349_s_at:2119:ETV5 | GSE23400(GPL96):211744_s_at:965:CD58* | GSE23400(GPL96):209291_at:3400:ID4 | GSE23400(GPL96):207186_s_at:2186:BPTF | GSE29001:206896_s_at:2788:GNG7 | -2.12E-11 |
| 29 | GSE23400(GPL97):226997_at:81792:ADAMTS12 | GSE23400(GPL96):214454_at:9509:ADAMTS2* | GSE20347:201438_at:1293:COL6A3* | GSE23400(GPL96):202598_at:6284:S100A13* | GSE23400(GPL97):230521_at:84904:C9orf100* | GSE20347:211022_s_at:546:ATRX* | GSE20347:202327_s_at:5310:PKD1* | GSE20347:217410_at:375790:AGRN* | -3.80E-11 |
| 30 | GSE29001:201852_x_at:1281:COL3A1 | GSE23400(GPL97):242654_at:2176:FANCC | GSE23400(GPL96):210220_at:2535:FZD2* | GSE20347:218618_s_at:64778:FNDC3B | GSE29001:213546_at:222161:DKFZP586I1420* | GSE23400(GPL97):234939_s_at:57649:PHF12* | GSE20347:206924_at:3589:IL11* | GSE23400(GPL97):227942_s_at:9419:CRIPT | -3.77E-11 |
| 31 | GSE20347:219997_s_at:64708:COPS7B | GSE20347:208770_s_at:1979:EIF4EBP2 | GSE23400(GPL97):232888_at:140862:ISM1* | GSE23400(GPL97):223843_at:51435:SCARA3 | GSE23400(GPL97):232458_at:1281:COL3A1 | GSE20347:201849_at:664:BNIP3* | GSE23400(GPL96):220840_s_at:55732:C1orf112* | GSE29001:209553_at:23355:VPS8 | -3.93E-11 |
| 32 | GSE23400(GPL96):209927_s_at:26097:C1orf77* | GSE20347:205430_at:653:BMP5* | GSE20347:212679_at:26608:TBL2* | GSE23400(GPL96):212344_at:23213:SULF1* | GSE29001:208202_s_at:23338:PHF15 | GSE29001:217875_s_at:56937:PMEPA1 | GSE23400(GPL97):227143_s_at:637:BID | GSE23400(GPL97):233848_x_at:7638:ZNF221 | -3.67E-11 |
| 33 | GSE29001:201852_x_at:1281:COL3A1 | GSE23400(GPL96):203264_s_at:23229:ARHGEF9* | GSE23400(GPL96):206086_x_at:3077:HFE | GSE23400(GPL97):231534_at:983:CDC2 | GSE20347:214341_at:79178:THTPA* | GSE23400(GPL97):233982_x_at:51657:STYXL1 | GSE23400(GPL96):203917_at:1525:CXADR* | GSE20347:206364_at:9928:KIF14 | -3.17E-11 |
| 34 | GSE23400(GPL96):217875_s_at:56937:PMEPA1 | GSE20347:202251_at:9129:PRPF3* | GSE23400(GPL96):222044_at:63935:PCIF1* | GSE20347:218599_at:9985:REC8 | GSE23400(GPL96):210581_x_at:23598:PATZ1* | GSE29001:221796_at:4915:NTRK2 | GSE20347:204007_at:2215:FCGR3B* | GSE29001:206321_at:5989:RFX1 | -7.38E-11 |
| 35 | GSE23400(GPL96):215468_at:647070:LOC647070* | GSE23400(GPL96):211725_s_at:637:BID | GSE23400(GPL97):240686_x_at:7037:TFRC | GSE20347:200892_s_at:6434:SFRS10* | GSE23400(GPL97):225258_at:54751:FBLIM1* | GSE23400(GPL96):213057_at:91647:ATPAF2* | GSE23400(GPL97):226823_at:65979:PHACTR4 | GSE29001:210737_at:7275:TUB | -1.59759704 |
| 36 | GSE20347:202635_s_at:5440:POLR2K* | GSE20347:204302_s_at:9811:KIAA0427* | GSE20347:213790_at:8038:ADAM12* | GSE23400(GPL96):207855_s_at:23155:CLCC1 | GSE23400(GPL97):242029_at:64778:FNDC3B | GSE20347:216415_at:55567:DNAH3* | GSE23400(GPL97):223451_s_at:51192:CKLF | GSE23400(GPL96):204645_at:905:CCNT2* | -1.04E-06 |
| 37 | GSE23400(GPL96):204320_at:1301:COL11A1 | GSE20347:205639_at:313:AOAH* | GSE20347:218562_s_at:55219:TMEM57 | GSE29001:210289_at:9027:NAT8* | GSE29001:219556_at:80178:C16orf59* | GSE29001:204691_x_at:8398:PLA2G6* | GSE23400(GPL97):229386_at:3400:ID4 | GSE23400(GPL97):225418_at:5819:PVRL2 | -5.29E-11 |
| 38 | GSE20347:218155_x_at:55720:TSR1 | GSE20347:214987_at:2549:GAB1 | GSE29001:219233_s_at:55876:GSDMB | GSE20347:201807_at:9559:VPS26A | GSE23400(GPL96):209362_at:9412:MED21* | GSE20347:218913_s_at:51291:GMIP | GSE29001:207173_x_at:1009:CDH11 | GSE23400(GPL97):222611_s_at:55269:PSPC1 | -6.79E-08 |
| 39 | GSE23400(GPL97):229218_at:1278:COL1A2 | GSE20347:219690_at:79713:TMEM149* | GSE23400(GPL97):226700_at:199746:U2AF1L4* | GSE29001:219418_at:79840:NHEJ1* | GSE23400(GPL96):220420_at:79748:LMAN1L* | GSE23400(GPL96):207326_at:685:BTC | GSE20347:213235_at:400506:C16orf88* | GSE29001:209464_at:9212:AURKB | -1.02E-10 |
| 40 | GSE23400(GPL96):221168_at:59336:PRDM13* | GSE20347:214840_at:146691:TOM1L2 | GSE20347:220825_s_at:55243:KIRREL | GSE23400(GPL96):216522_at:26212:OR2B6* | GSE29001:222374_at:8945:BTRC | GSE20347:202704_at:10140:TOB1 | GSE20347:220923_s_at:29944:PNMA3 | GSE23400(GPL97):235908_at:4320:MMP11 | -2.52E-11 |
| 41 | GSE23400(GPL97):229865_at:64778:FNDC3B | GSE23400(GPL96):209926_at:4207:MEF2B* | GSE20347:210130_s_at:7108:TM7SF2* | GSE23400(GPL97):229246_at:645460:FLJ44342* | GSE23400(GPL96):204320_at:1301:COL11A1 | GSE23400(GPL97):231083_at:2119:ETV5 | GSE20347:212283_at:375790:AGRN* | GSE23400(GPL97):225375_at:147007:TMEM199* | -5.98E-11 |
| 42 | GSE23400(GPL96):208767_s_at:55353:LAPTM4B* | GSE23400(GPL97):243623_at:6512:SLC1A7 | GSE29001:40524_at:11099:PTPN21 | GSE29001:209680_s_at:3833:KIFC1* | GSE20347:203349_s_at:2119:ETV5 | GSE23400(GPL97):222581_at:9213:XPR1* | GSE23400(GPL96):205910_s_at:1056:CEL* | GSE29001:213375_s_at:90634:N4BP2L1 | -0.438953419 |
| 43 | GSE29001:203878_s_at:4320:MMP11 | GSE20347:202128_at:9870:KIAA0317 | GSE23400(GPL96):204188_s_at:5916:RARG* | GSE29001:206441_s_at:54939:COMMD4* | GSE29001:205393_s_at:1111:CHEK1 | GSE20347:218905_at:55656:INTS8* | GSE23400(GPL97):224859_at:80381:CD276* | GSE23400(GPL97):237866_at:55022:PID1 | -1.47E-04 |
| 44 | GSE29001:204849_at:10732:TCFL5 | GSE29001:201944_at:3074:HEXB* | GSE29001:209292_at:3400:ID4 | GSE23400(GPL97):232969_at:22900:CARD8 | GSE23400(GPL96):202417_at:9817:KEAP1* | GSE23400(GPL96):216375_s_at:2119:ETV5 | GSE20347:222200_s_at:55108:BSDC1* | GSE29001:204063_s_at:9706:ULK2* | -6.20E-11 |
| 45 | GSE20347:202233_s_at:7388:UQCRH* | GSE29001:211571_s_at:1462:VCAN* | GSE23400(GPL97):224400_s_at:83539:CHST9* | GSE23400(GPL97):226598_s_at:26164:GTPBP5* | GSE20347:211725_s_at:637:BID | GSE29001:201720_s_at:7805:LAPTM5* | GSE29001:218875_s_at:26271:FBXO5 | GSE20347:201939_at:10769:PLK2* | -0.001649284 |
| 46 | GSE23400(GPL97):226344_at:84460:ZMAT1* | GSE20347:202403_s_at:1278:COL1A2 | GSE29001:204644_at:10495:ENOX2 | GSE23400(GPL96):203764_at:9787:DLGAP5* | GSE23400(GPL96):218618_s_at:64778:FNDC3B | GSE23400(GPL96):204914_s_at:6664:SOX11* | GSE23400(GPL96):203744_at:3149:HMGB3 | GSE29001:206319_s_at:57119:SPINLW1* | -4.90E-11 |
| 47 | GSE23400(GPL97):228358_at:6666:SOX12 | GSE29001:203086_at:3796:KIF2A* | GSE20347:207362_at:7782:SLC30A4 | GSE23400(GPL96):209291_at:3400:ID4 | GSE29001:218305_at:79711:IPO4* | GSE20347:204849_at:10732:TCFL5 | GSE20347:212114_at:552889:LOC552889* | GSE20347:207172_s_at:1009:CDH11 | -2.88E-11 |
| 48 | GSE29001:205161_s_at:8800:PEX11A* | GSE23400(GPL97):227155_at:8543:LMO4 | GSE20347:215458_s_at:57154:SMURF1* | GSE23400(GPL97):223675_s_at:55591:VEZT | GSE20347:217430_x_at:1277:COL1A1* | GSE23400(GPL97):228774_at:84131:CEP78* | GSE29001:209291_at:3400:ID4 | GSE23400(GPL97):230500_at:5150:PDE7A* | -6.80E-07 |
| Each row represents an improved *c-LM*. Each *AB* presenting in each cell is composed of GEO accession number of dataset (platform), probe ID, gene ID and gene symbol. #: Serial number of models; *: non-common genes; *ABs*: associated biomarkers; *k*: Number of *ABs*; *LLV*: natural log likelihood of an improved *c-LM*. | | | | | | | | | |

**Table S2.** List of 48 improved c-*LMs* (*k*=8) trained from all datasets in HCC input set.

| # | 1^st^ *AB* | 2^nd^ *AB* | 3^rd^ *AB* | 4^th^ *AB* | 5^th^ *AB* | 6^th^ *AB* | 7^th^ *AB* | 8^th^ *AB* | *LLV* |
| --- | --- | --- | --- | --- | --- | --- | --- | --- | --- |
| 1 | GSE14520(GPL3921):202752_x_at:23428:SLC7A8 | GSE14520(GPL571):218061_at:4201:MEA1 | GSE14520(GPL3921):220114_s_at:55576:STAB2 | GSE17856:A_23_P132260:23481:PES1 | GSE17856:A_23_P108994:6185:RPN2 | GSE14520(GPL3921):206551_x_at:54800:KLHL24 | GSE17856:A_24_P303989:648:BMI1 | GSE17856:A_23_P103837:9898:UBAP2L | -11.75 |
| 2 | GSE17856:A_23_P144872:2760:GM2A | GSE17856:A_23_P118266:463:ATBF1 | GSE14520(GPL571):201469_s_at:6464:SHC1 | GSE14520(GPL571):210457_x_at:3159:HMGA1 | GSE14520(GPL571):209188_x_at:1810:DR1 | GSE14520(GPL571):218002_s_at:9547:CXCL14 | GSE14520(GPL3921):212544_at:9326:ZNHIT3 | GSE17856:A_24_P373174:5873:RAB27A | -13.1 |
| 3 | GSE14520(GPL571):202186_x_at:5525:PPP2R5A | GSE14520(GPL571):216903_s_at:10367:MICU1 | GSE17856:A_32_P116556:84627:ZNF469* | GSE14520(GPL3921):221923_s_at:4869:NPM1 | GSE14520(GPL3921):219541_at:54923:LIME1 | GSE14520(GPL571):202276_at:7979:SHFM1 | GSE14520(GPL571):210720_s_at:63941:APBA2BP | GSE14520(GPL3921):211156_at:1029:CDKN2A | -9.07 |
| 4 | GSE14520(GPL571):209504_s_at:58473:PLEKHB1* | GSE14520(GPL571):209116_x_at:3043:HBB | GSE14520(GPL571):201293_x_at:5478:PPIA | GSE14520(GPL3921):204866_at:9767:PHF16 | GSE17856:A_23_P115732:648:BMI1 | GSE17856:A_23_P321949:5320:PLA2G2A | GSE14520(GPL571):208937_s_at:3397:ID1* | GSE14520(GPL3921):205449_at:29901:SAC3D1 | -8.55 |
| 5 | GSE17856:A_23_P106194:2353:FOS | GSE14520(GPL3921):212143_s_at:3486:IGFBP3 | GSE14520(GPL3921):205446_s_at:1386:ATF2 | GSE14520(GPL3921):201293_x_at:5478:PPIA | GSE14520(GPL3921):214925_s_at:6709:SPTAN1 | GSE14520(GPL3921):219569_s_at:80723:TMEM22 | GSE14520(GPL571):204011_at:10253:SPRY2 | GSE14520(GPL3921):220946_s_at:29072:SETD2 | -10.23 |
| 6 | GSE17856:A_24_P342807:55676:SLC30A6* | GSE17856:A_23_P102582:55969:C20orf24 | GSE17856:A_32_P867789:57642:COL20A1* | GSE14520(GPL571):209365_s_at:1893:ECM1 | GSE17856:A_23_P100111:11261:CHP | GSE14520(GPL3921):210481_s_at:10332:CLEC4M | GSE14520(GPL571):221932_s_at:51218:GLRX5 | GSE14520(GPL571):218482_at:56943:ENY2 | -11.82 |
| 7 | GSE14520(GPL3921):204192_at:951:CD37 | GSE14520(GPL3921):217956_s_at:58478:MASA | GSE14520(GPL3921):201275_at:2224:FDPS | GSE17856:A_23_P206110:1544:CYP1A2 | GSE17856:A_23_P130753:1628:DBP | GSE14520(GPL3921):213869_x_at:7070:THY1 | GSE14520(GPL3921):202710_at:10282:BET1 | GSE17856:A_23_P213745:9547:CXCL14 | -14.06 |
| 8 | GSE17856:A_32_P99432:126003:TRAPPC5* | GSE17856:A_23_P90510:92840:REEP6* | GSE14520(GPL3921):200043_at:2079:ERH | GSE14520(GPL3921):208149_x_at:1663:DDX11 | GSE17856:A_23_P160559:1893:ECM1 | GSE14520(GPL571):210235_s_at:8500:PPFIA1 | GSE17856:A_24_P141005:6856:SYPL1 | GSE17856:A_24_P535256:3624:INHBA | -11.4 |
| 9 | GSE14520(GPL571):218378_s_at:79706:PRKRIP1 | GSE14520(GPL3921):216652_s_at:1810:DR1 | GSE14520(GPL571):216589_at:390998:LOC390998* | GSE14520(GPL571):200701_at:10577:NPC2* | GSE17856:A_23_P401:1063:CENPF | GSE14520(GPL3921):214244_s_at:8992:ATP6V0E1 | GSE17856:A_23_P164057:4239:MFAP4 | GSE14520(GPL3921):210481_s_at:10332:CLEC4M | -10.56 |
| 10 | GSE14520(GPL3921):214420_s_at:1559:CYP2C9* | GSE14520(GPL3921):211060_x_at:8733:GPAA1 | GSE14520(GPL571):211760_s_at:8674:VAMP4 | GSE17856:A_23_P201376:117178:SSX2IP | GSE14520(GPL3921):212554_at:10486:CAP2 | GSE14520(GPL571):203316_s_at:6635:SNRPE | GSE17856:A_23_P131676:57007:CXCR7 | GSE14520(GPL571):203355_s_at:23362:PSD3 | -14.32 |
| 11 | GSE14520(GPL3921):215424_s_at:22938:SNW1 | GSE14520(GPL571):211696_x_at:3043:HBB | GSE14520(GPL3921):207259_at:55018:LINC00483 | GSE14520(GPL571):218011_at:59286:UBL5 | GSE14520(GPL3921):206007_at:10216:PRG4 | GSE17856:A_23_P61569:8674:VAMP4 | GSE14520(GPL571):201293_x_at:5478:PPIA | GSE14520(GPL571):218002_s_at:9547:CXCL14 | -10.77 |
| 12 | GSE17856:A_23_P138541:8644:AKR1C3 | GSE14520(GPL3921):207218_at:2158:F9* | GSE14520(GPL3921):218285_s_at:56898:BDH2 | GSE17856:A_23_P131723:10971:YWHAQ | GSE14520(GPL571):214512_s_at:10923:SUB1 | GSE14520(GPL571):208439_s_at:2220:FCN2* | GSE17856:A_23_P29495:1499:CTNNB1 | GSE14520(GPL3921):214114_x_at:10922:FASTK | -8.81 |
| 13 | GSE17856:A_23_P47839:57696:DDX55* | GSE17856:A_23_P52639:1351:COX8A | GSE17856:A_23_P82979:10319:LAMC3 | GSE14520(GPL571):207995_s_at:10332:CLEC4M | GSE17856:A_23_P348298:29901:SAC3D1 | GSE14520(GPL571):211156_at:1029:CDKN2A | GSE14520(GPL571):214114_x_at:10922:FASTK | GSE17856:A_24_P180654:64764:CREB3L2 | -8.86 |
| 14 | GSE14520(GPL571):200842_s_at:2058:EPRS | GSE14520(GPL571):217889_s_at:79901:CYBRD1 | GSE14520(GPL3921):203272_s_at:11334:TUSC2 | GSE14520(GPL3921):218002_s_at:9547:CXCL14 | GSE17856:A_23_P258340:5478:PPIA | GSE14520(GPL3921):222005_s_at:2785:GNG3* | GSE14520(GPL3921):217641_at:64582:GPR135 | GSE14520(GPL571):201656_at:3655:ITGA6 | -9.08 |
| 15 | GSE17856:A_23_P162607:55576:STAB2 | GSE14520(GPL571):215138_s_at:23254:KAZN | GSE14520(GPL571):203109_at:9040:UBE2M | GSE17856:A_32_P435367:5710:PSMD4 | GSE14520(GPL3921):206261_at:8187:ZNF239 | GSE17856:A_23_P21033:8833:GMPS | GSE17856:A_24_P823011:9949:AMMECR1 | GSE14520(GPL3921):221018_s_at:56165:TDRD1* | -13.18 |
| 16 | GSE14520(GPL571):203194_s_at:4928:NUP98 | GSE14520(GPL3921):212448_at:23327:NEDD4L | GSE14520(GPL3921):209687_at:6387:CXCL12 | GSE17856:A_24_P376556:54205:CYCS | GSE14520(GPL571):202813_at:6894:TARBP1 | GSE17856:A_24_P340679:5478:PPIA | GSE14520(GPL3921):218213_s_at:746:C11orf10 | GSE17856:A_23_P422178:8450:CUL4B | -8.14 |
| 17 | GSE14520(GPL571):201359_at:1315:COPB1 | GSE14520(GPL3921):201591_s_at:11188:NISCH | GSE14520(GPL571):213194_at:6091:ROBO1 | GSE14520(GPL3921):209365_s_at:1893:ECM1 | GSE17856:A_23_P132139:54058:C21orf58* | GSE14520(GPL3921):221747_at:7145:TNS1 | GSE14520(GPL3921):203493_s_at:9702:CEP57 | GSE14520(GPL3921):200805_at:10960:LMAN2 | -11.69 |
| 18 | GSE14520(GPL571):219256_s_at:54436:SH3TC1 | GSE14520(GPL571):212137_at:23367:LARP1 | GSE14520(GPL3921):213649_at:6432:SRSF7 | GSE17856:A_23_P158148:908:CCT6A | GSE17856:A_23_P202029:10613:SPFH1 | GSE14520(GPL571):201075_s_at:6599:SMARCC1 | GSE14520(GPL571):202276_at:7979:SHFM1 | GSE17856:A_23_P52639:1351:COX8A | -10.33 |
| 19 | GSE17856:A_32_P148345:302:ANXA2 | GSE17856:A_32_P206698:1163:CKS1B | GSE14520(GPL571):206531_at:8193:DPF1* | GSE17856:A_23_P33444:6645:SNTB2 | GSE17856:A_23_P213745:9547:CXCL14 | GSE14520(GPL571):205661_s_at:80308:FLAD1 | GSE17856:A_32_P222521:9604:RNF14 | GSE14520(GPL571):215455_at:8914:TIMELESS | -11.42 |
| 20 | GSE17856:A_24_P250922:5743:PTGS2 | GSE14520(GPL3921):40562_at:2767:GNA11 | GSE14520(GPL571):208806_at:1107:CHD3 | GSE14520(GPL3921):201656_at:3655:ITGA6 | GSE14520(GPL3921):205044_at:2568:GABRP | GSE14520(GPL571):211978_x_at:5478:PPIA | GSE14520(GPL3921):218289_s_at:79876:UBE1DC1 | GSE14520(GPL571):202873_at:528:ATP6V1C1 | -8.38 |
| 21 | GSE14520(GPL3921):219059_s_at:10894:XLKD1* | GSE14520(GPL3921):218002_s_at:9547:CXCL14 | GSE14520(GPL571):208228_s_at:2263:FGFR2 | GSE14520(GPL3921):209194_at:1069:CETN2 | GSE14520(GPL3921):201261_x_at:633:BGN | GSE14520(GPL3921):206929_s_at:4782:NFIC | GSE17856:A_23_P70127:54732:TMED9 | GSE17856:A_23_P421664:10486:CAP2 | -8.38 |
| 22 | GSE14520(GPL3921):219628_at:64393:ZMAT3 | GSE17856:A_23_P10911:196463:PLBD2* | GSE14520(GPL571):214244_s_at:8992:ATP6V0E1 | GSE14520(GPL3921):211765_x_at:5478:PPIA | GSE14520(GPL571):218002_s_at:9547:CXCL14 | GSE17856:A_23_P108303:4701:NDUFA7 | GSE17856:A_23_P125056:575:BAI1 | GSE14520(GPL3921):214414_x_at:3040:HBA2* | -6.18 |
| 23 | GSE17856:A_24_P414712:27154:BRPF3* | GSE14520(GPL571):215815_at:8896:BUD31 | GSE17856:A_24_P153853:4591:TRIM37 | GSE14520(GPL3921):207995_s_at:10332:CLEC4M | GSE14520(GPL3921):219371_s_at:10365:KLF2 | GSE14520(GPL571):206338_at:1995:ELAVL3* | GSE14520(GPL571):218417_s_at:55652:FLJ20489 | GSE17856:A_23_P125171:2272:FHIT | -12.64 |
| 24 | GSE14520(GPL571):206492_at:2272:FHIT | GSE17856:A_24_P544882:6745:SSR1 | GSE14520(GPL3921):37433_at:9063:PIAS2 | GSE14520(GPL3921):203397_s_at:2591:GALNT3 | GSE17856:A_23_P254573:27000:ZRF1 | GSE14520(GPL3921):211745_x_at:3039:HBA1* | GSE14520(GPL3921):208757_at:54732:TMED9 | GSE14520(GPL571):211765_x_at:5478:PPIA | -8.18 |
| 25 | GSE14520(GPL3921):218887_at:51069:MRPL2 | GSE14520(GPL571):207609_s_at:1544:CYP1A2 | GSE14520(GPL571):202213_s_at:8450:CUL4B | GSE14520(GPL571):219293_s_at:29789:GTPBP9 | GSE14520(GPL571):205849_s_at:7381:UQCRB | GSE14520(GPL571):217917_s_at:83658:DYNLRB1 | GSE14520(GPL571):202276_at:7979:SHFM1 | GSE14520(GPL3921):218667_at:64219:PJA1 | -9.93 |
| 26 | GSE17856:A_32_P6917:80308:FLAD1 | GSE14520(GPL571):206960_at:2846:GPR23* | GSE14520(GPL571):212067_s_at:715:C1R | GSE14520(GPL571):209142_s_at:7326:UBE2G1 | GSE14520(GPL571):206459_s_at:7482:WNT2B* | GSE14520(GPL3921):212551_at:10486:CAP2 | GSE14520(GPL3921):221747_at:7145:TNS1 | GSE14520(GPL571):203484_at:23480:SEC61G | -9.86 |
| 27 | GSE14520(GPL3921):202961_s_at:9551:ATP5J2 | GSE14520(GPL3921):210844_x_at:1495:CTNNA1 | GSE14520(GPL3921):201180_s_at:2773:GNAI3 | GSE17856:A_23_P502142:2534:FYN | GSE17856:A_23_P25515:9107:MTMR6 | GSE17856:A_23_P151405:26586:CKAP2 | GSE14520(GPL3921):65472_at:10713:USP39 | GSE14520(GPL571):207039_at:1029:CDKN2A | -7.87 |
| 28 | GSE17856:A_23_P348298:29901:SAC3D1 | GSE14520(GPL571):33646_g_at:2760:GM2A | GSE17856:A_23_P254573:27000:ZRF1 | GSE14520(GPL3921):215987_at:9693:RAPGEF2 | GSE17856:A_23_P374389:170394:PWWP2B* | GSE17856:A_23_P138541:8644:AKR1C3 | GSE14520(GPL3921):200820_at:5714:PSMD8 | GSE17856:A_24_P344961:154796:AMOT | -10.98 |
| 29 | GSE17856:A_24_P84099:387885:CCDC42B* | GSE14520(GPL3921):201575_at:22938:SNW1 | GSE17856:A_23_P25073:60488:MRPS35 | GSE14520(GPL571):220249_at:23553:HYAL4* | GSE14520(GPL3921):220037_s_at:10894:XLKD1* | GSE14520(GPL571):203666_at:6387:CXCL12 | GSE14520(GPL571):218188_s_at:26517:TIMM13 | GSE17856:A_24_P920125:5478:PPIA | -9.26 |
| 30 | GSE14520(GPL571):221470_s_at:27178:IL37 | GSE17856:A_32_P125496:374354:NHLRC2 | GSE14520(GPL3921):210720_s_at:63941:APBA2BP | GSE14520(GPL571):200693_at:10971:YWHAQ | GSE14520(GPL3921):219293_s_at:29789:GTPBP9 | GSE14520(GPL571):212554_at:10486:CAP2 | GSE14520(GPL3921):207142_at:3760:KCNJ3* | GSE17856:A_23_P20558:55664:CDC37L1 | -11.16 |
| 31 | GSE14520(GPL571):209189_at:2353:FOS | GSE17856:A_32_P121651:57534:MIB1* | GSE14520(GPL3921):205448_s_at:7786:MAP3K12 | GSE17856:A_23_P126291:6635:SNRPE | GSE14520(GPL3921):208711_s_at:595:CCND1 | GSE14520(GPL571):209391_at:8818:DPM2 | GSE17856:A_23_P258340:5478:PPIA | GSE17856:A_23_P66787:47:ACLY | -9.99 |
| 32 | GSE14520(GPL571):204950_at:22900:CARD8 | GSE17856:A_32_P170444:10923:SUB1 | GSE14520(GPL571):205423_at:162:AP1B1 | GSE14520(GPL3921):211978_x_at:5478:PPIA | GSE14520(GPL3921):208825_x_at:6147:RPL23A | GSE17856:A_23_P115316:64222:TOR3A | GSE14520(GPL571):209687_at:6387:CXCL12 | GSE17856:A_23_P52639:1351:COX8A | -11.08 |
| 33 | GSE17856:A_24_P106953:80142:PTGES2 | GSE14520(GPL571):205777_at:1852:DUSP9 | GSE14520(GPL571):218281_at:51642:MRPL48 | GSE14520(GPL3921):212763_at:23271:CAMSAP2 | GSE14520(GPL3921):207609_s_at:1544:CYP1A2 | GSE17856:A_23_P134650:26024:PTCD1 | GSE14520(GPL3921):211378_x_at:5478:PPIA | GSE14520(GPL3921):215268_at:643314:KIAA0754* | -9.26 |
| 34 | GSE17856:A_24_P320328:10923:SUB1 | GSE17856:A_23_P47247:55346:TCP11L1 | GSE14520(GPL571):201622_at:27044:SND1 | GSE14520(GPL3921):217942_at:60488:MRPS35 | GSE17856:A_24_P173124:201163:FLCN | GSE14520(GPL571):211765_x_at:5478:PPIA | GSE14520(GPL3921):203666_at:6387:CXCL12 | GSE17856:A_32_P300427:164284:APCDD1L* | -9.28 |
| 35 | GSE14520(GPL571):220331_at:10858:CYP46A1* | GSE17856:A_23_P99930:54962:TIPIN | GSE14520(GPL3921):209365_s_at:1893:ECM1 | GSE14520(GPL571):201671_x_at:9097:USP14 | GSE14520(GPL571):209284_s_at:23272:FAM208A | GSE17856:A_23_P348298:29901:SAC3D1 | GSE14520(GPL3921):211997_x_at:3021:H3F3B* | GSE17856:A_23_P90444:79171:MGC10433 | -11.94 |
| 36 | GSE14520(GPL571):210892_s_at:2969:GTF2I | GSE14520(GPL3921):207127_s_at:3189:HNRPH3 | GSE17856:A_24_P410657:91368:CDKN2AIPNL* | GSE14520(GPL3921):219275_at:9141:PDCD5 | GSE14520(GPL571):204201_s_at:5783:PTPN13 | GSE14520(GPL3921):210481_s_at:10332:CLEC4M | GSE17856:A_23_P56091:126259:TMIGD2* | GSE14520(GPL3921):200932_s_at:10540:DCTN2 | -10.41 |
| 37 | GSE14520(GPL571):216589_at:390998:LOC390998* | GSE14520(GPL571):219638_at:26263:FBXO22 | GSE14520(GPL571):203316_s_at:6635:SNRPE | GSE14520(GPL3921):213490_s_at:5605:MAP2K2 | GSE14520(GPL571):210506_at:2529:FUT7* | GSE14520(GPL3921):212232_at:23360:FNBP4 | GSE14520(GPL571):203183_s_at:6602:SMARCD1 | GSE17856:A_23_P406424:389:RHOC | -9.72 |
| 38 | GSE17856:A_24_P254346:10311:DSCR3 | GSE14520(GPL3921):218132_s_at:79042:TSEN34* | GSE14520(GPL571):204871_at:7978:MTERF | GSE17856:A_23_P74162:55721:IQCC | GSE14520(GPL571):209365_s_at:1893:ECM1 | GSE14520(GPL3921):201127_s_at:47:ACLY | GSE14520(GPL571):215443_at:7253:TSHR* | GSE14520(GPL571):203235_at:7064:THOP1 | -10.07 |
| 39 | GSE14520(GPL571):218391_at:11267:SNF8 | GSE17856:A_24_P256674:9639:ARHGEF10 | GSE14520(GPL571):218356_at:29960:FTSJ2 | GSE14520(GPL571):218002_s_at:9547:CXCL14 | GSE14520(GPL571):212949_at:23397:NCAPH | GSE14520(GPL571):213003_s_at:1052:CEBPD | GSE14520(GPL571):218514_at:55181:SMG8 | GSE17856:A_23_P43484:1029:CDKN2A | -10.16 |
| 40 | GSE17856:A_23_P80778:84925:DIRC2* | GSE14520(GPL3921):205044_at:2568:GABRP | GSE14520(GPL3921):201128_s_at:47:ACLY | GSE14520(GPL571):212247_at:23165:NUP205 | GSE14520(GPL3921):221923_s_at:4869:NPM1 | GSE14520(GPL3921):220496_at:51266:CLEC1B* | GSE17856:A_23_P68628:63941:APBA2BP | GSE14520(GPL571):203951_at:1264:CNN1 | -10.8 |
| 41 | GSE14520(GPL3921):215752_at:23235:SNF1LK2 | GSE17856:A_23_P414343:4496:MT1H | GSE14520(GPL571):214102_at:116984:CENTD1 | GSE14520(GPL3921):209478_at:5098:PCDHGC3* | GSE14520(GPL571):202186_x_at:5525:PPP2R5A | GSE14520(GPL571):201127_s_at:47:ACLY | GSE14520(GPL571):202475_at:10430:TMEM147 | GSE17856:A_23_P43484:1029:CDKN2A | -6.65 |
| 42 | GSE14520(GPL571):218370_s_at:64766:S100PBP | GSE14520(GPL3921):210543_s_at:5591:PRKDC | GSE14520(GPL571):219711_at:54807:ZNF586 | GSE17856:A_23_P30745:4201:MEA1 | GSE14520(GPL571):212551_at:10486:CAP2 | GSE14520(GPL3921):40020_at:1951:CELSR3* | GSE14520(GPL571):209644_x_at:1029:CDKN2A | GSE14520(GPL3921):214278_s_at:57447:NDRG2 | -8.88 |
| 43 | GSE17856:A_32_P229746:10049:DNAJB6 | GSE14520(GPL3921):210372_s_at:7164:TPD52L1 | GSE14520(GPL3921):205019_s_at:7433:VIPR1* | GSE14520(GPL571):200774_at:23196:FAM120A | GSE14520(GPL571):219774_at:54520:CCDC93 | GSE14520(GPL571):221016_s_at:83439:TCF7L1 | GSE14520(GPL3921):214563_at:5098:PCDHGC3* | GSE14520(GPL571):214773_x_at:261726:TIPRL | -10.63 |
| 44 | GSE14520(GPL3921):203114_at:10534:SSSCA1* | GSE14520(GPL571):206452_x_at:5524:PPP2R4 | GSE17856:A_23_P159797:8228:PNPLA4 | GSE14520(GPL571):209189_at:2353:FOS | GSE14520(GPL571):217021_at:1528:CYB5A* | GSE14520(GPL3921):212554_at:10486:CAP2 | GSE14520(GPL3921):201577_at:4830:NME1* | GSE14520(GPL571):210720_s_at:63941:APBA2BP | -8.77 |
| 45 | GSE14520(GPL3921):209754_s_at:7112:TMPO | GSE14520(GPL3921):202861_at:5187:PER1 | GSE14520(GPL571):208815_x_at:3308:HSPA4 | GSE14520(GPL571):203816_at:1716:DGUOK | GSE14520(GPL3921):211627_x_at:2099:ESR1 | GSE14520(GPL571):213336_at:9031:BAZ1B | GSE14520(GPL3921):213390_at:23211:C19orf7 | GSE14520(GPL3921):206680_at:922:CD5L | -16.05 |
| 46 | GSE17856:A_23_P160559:1893:ECM1 | GSE14520(GPL571):210543_s_at:5591:PRKDC | GSE17856:A_23_P427039:140838:NANP* | GSE17856:A_24_P339611:9141:PDCD5 | GSE14520(GPL571):218185_s_at:55156:ARMC1 | GSE17856:A_24_P214598:152926:PPM1K* | GSE17856:A_23_P206110:1544:CYP1A2 | GSE17856:A_23_P20045:5189:PEX1 | -11.55 |
| 47 | GSE14520(GPL3921):209489_at:10658:CELF1 | GSE14520(GPL3921):200693_at:10971:YWHAQ | GSE17856:A_23_P137848:79590:MRPL24 | GSE14520(GPL3921):204383_at:8220:DGCR14 | GSE14520(GPL571):218550_s_at:55222:LRRC20 | GSE14520(GPL571):201293_x_at:5478:PPIA | GSE14520(GPL3921):218233_s_at:29964:C6orf49* | GSE17856:A_23_P90510:92840:REEP6* | -10.15 |
| 48 | GSE14520(GPL571):210720_s_at:63941:APBA2BP | GSE17856:A_24_P302406:23261:CAMTA1 | GSE14520(GPL571):220120_s_at:64097:EPB41L4A* | GSE14520(GPL3921):221325_at:56659:KCNK13* | GSE14520(GPL3921):200021_at:1072:CFL1 | GSE14520(GPL3921):205165_at:1951:CELSR3* | GSE14520(GPL571):202690_s_at:6632:SNRPD1 | GSE17856:A_23_P386450:51426:POLK* | -8.8 |
| Each row represents an improved *c-LM*. Each *AB* presenting in each cell is composed of GEO accession number of dataset (platform), probe ID, gene ID and gene symbol. #: Serial number of models; *: non-common genes; *ABs*: associated biomarkers; *k*: Number of *ABs*; *LLV*: natural log likelihood of an improved *c-LM*. | | | | | | | | | |
