# Supplementary material for: MiningABs: mining associated biomarkers across multi-connected gene expression datasets
Source: BMC Bioinformatics. 2014 Jun 8;15:173. doi: 10.1186/1471-2105-15-173 (PMC4068973; doi:10.1186/1471-2105-15-173)
Supplement: Additional file 2 — List of ABs involved in cancer-related GO terms for the ESCC and HCC input sets. [file 1471-2105-15-173-S2.doc]

**Table S3.** List of *ABs* involved in cancer-related GO terms for the ESCC input set.

| Serial # | GO terms | ABs |
| --- | --- | --- |
| 1 | DNA replication | CHAF1A,LOC642954,RBBP4,LOC648152,LOC651921,ATR,CHEK1,NFIB,LOC100130009,HMGA1,Cdc45,sirt1 |
| 2 | Negative regulation of biological process | Runx1,SMURF1,HMGB3,LOC729595,LOC646993,LOC729952,BTC,rarg,UBE2I,Aoah,Arf6,Cdc45,BNIP3,Cdk1,CD276,cxadr,CXADRP2,CARD8,thrA,Fst,Eif4ebp2,NFIB,MMP14,ABHD2,Tob1,COL3A1,foxo1,LOC642954,RBBP4,Rgs5,BAX,CHEK1,CHST11,LOC100130009,HMGA1,Fbxo5,PHF12,CASP2,B4GALT7,ZNF238,Igf2bp2,LOC648152,LOC651921,ATR,COL1A1,id4,BPTF,sirt1,IL11,cgrrf1 |
| 3 | Biological adhesion | Col6a3,COL3A1,Pkd1,CD58,COL11A1,CDH11,Arf6,adam12,C1orf38,cxadr,CXADRP2,ANTXR1,thbs2,thrA,PGM5,VCAN,DSC2,FBLIM1,SYMPK,PVRL2,DDR2 |
| 4 | Transmembrane receptor protein serine/threonine kinase signaling pathway | Tob1,COL3A1,SMURF1,Fst,GDF5,COL1A2 |
| 5 | Chromatin organization | CHAF1A,BMI1,LOC642954,RBBP4,safb,LOC100130009,HMGA1,BNIP3,Rtf1,Msl1,TLK2,Hist1h1t,Phf15,BPTF,sirt1 |
| 6 | DNA metabolic process | fanci,CHAF1A,Rec8,LOC642954,RBBP4,BAX,CHEK1,LOC100130009,HMGA1,Cdc45,BNIP3,Nhej1,TSN,Atrx,LOC648152,LOC651921,ATR,Fancc,NFIB,sirt1 |
| 7 | Chromosome organization | CHAF1A,Rec8,BMI1,LOC642954,RBBP4,KIFC1,safb,LOC100130009,HMGA1,BNIP3,Rtf1,Msl1,TLK2,Hist1h1t,DLGAP5,Phf15,BPTF,sirt1 |
| 8 | Regulation of mitochondrial membrane permeability | BID,BAX,BNIP3 |
| 9 | Mitosis | AURKB,Cdca3,KIF23,ZWILCH,KIFC1,UBE2I,DLGAP5,Fbxo5,CETN3,Cdk1 |
| 10 | Induction of apoptosis by extracellular signals | ECT2,BID,BAX,KALRN,LOC100134381,ARHGEF9,CASP2s |
| 11 | Cell adhesion | Pkd1,CD58,thrA,CDH11,COL11A1,DSC2,PVRL2 |
| 12 | M phase | AURKB,Rec8,Cdca3,KIF23,CHEK1,ZWILCH,KIFC1,UBE2I,DLGAP5,Fbxo5,CETN3,Cdk1 |
| 13 | Regulation of fibroblast proliferation | B4GALT7,BTC,BMI1,BAX |
| 14 | M phase of mitotic cell cycle | AURKB,Cdca3,KIF23,ZWILCH,KIFC1,UBE2I,DLGAP5,Fbxo5,CETN3,Cdk1 |
| 15 | Mitotic cell cycle | KIFC1,CHEK1,btrc,UBE2I,Fbxo5,CETN3,Cdk1,plk2,AURKB,Cdca3,KIF23,ZWILCH,id4,DLGAP5 |
| 16 | Regulation of cell differentiation | Runx1,S1PR5,Tob1,HLA-DOA,AGRN,HMGB3,LOC729595,LOC646993,LOC729952,BTC,BAX,keap1,GDF5,FNDC3B,CD276,Tcfl5,id4,Fst,NTRK2,KALRN,sirt1 |
| 17 | Cellular developmental process | Runx1,ABI2,S100A13,vamp5,SMURF1,HOXB7,Rec8,Robo4,adam12,BNIP3,Nhej1,ECE2,cxadr,CXADRP2,Tcfl5,Psme4,TRAPPC9,Hist1h1t,KIF2A,Fancc,VCAN,KALRN,SOX11,MMP14,S1PR5,ECT2,AGRN,BAX,COL11A1,CHST11,Fbxo5,BMP5,prdm13,Ulk2,PEX11A,FZD2,COL1A1,id4,ANTXR1,SEMA6D,NTRK2,PATZ1,Txnrd3,sirt1,PVRL2,IL11 |
| 18 | Cell cycle | fanci,Pkd1,Rec8,btrc,UBE2I,Cdc45,CETN3,Cdk1,TLK2,KIF23,Cdca3,CHAF1A,STRADA,LOC642954,RBBP4,KIFC1,CHEK1,Fbxo5,plk2,AURKB,LOC648152,LOC651921,ATR,id4,ZWILCH,DLGAP5,CCNT2,cgrrf1 |
| 19 | Cell differentiation | Runx1,ABI2,S100A13,vamp5,SMURF1,HOXB7,Rec8,Robo4,adam12,BNIP3,Nhej1,ECE2,cxadr,CXADRP2,Tcfl5,Psme4,TRAPPC9,Hist1h1t,KIF2A,Fancc,VCAN,KALRN,SOX11,MMP14,S1PR5,AGRN,BAX,COL11A1,CHST11,Fbxo5,BMP5,prdm13,Ulk2,PEX11A,FZD2,COL1A1,id4,ANTXR1,SEMA6D,NTRK2,PATZ1,Txnrd3,sirt1,PVRL2,IL11 |
| 20 | Developmental process | ABI2,Col6a3,vamp5,MEF2B,LOC729991-MEF2B,HMGB3,LOC729595,LOC646993,LOC729952,BMI1,rarg,CDH11,Arf6,MED21,Nhej1,TFEB,cxadr,CXADRP2,Hist1h1t,ADAMTS2,thrA,SOX11,ECT2,S1PR5,COL3A1,AGRN,Lmo4,TFRC,BAX,PRELP,SLC30A4,keap1,Ulk2,Atrx,LOC648152,LOC651921,ATR,id4,COL1A1,SEMA6D,COL1A2,Txnrd3,sirt1,IL11,EPOR,MMP11,Runx1,S100A13,Pkd1,HOXB7,SMURF1,Rec8,gss,Robo4,CA4,GDF5,BNIP3,adam12,PDE5A,Nat8,Nat8b,ECE2,Tcfl5,Psme4,TRAPPC9,Fst,KIF2A,Fancc,NFIB,VCAN,Hexb,KALRN,MMP14,foxo1,heyl,COL11A1,CHST11,Fbxo5,prdm13,BMP5,Igf2bp2,PEX11A,FZD2,ANTXR1,BPTF,NTRK2,PATZ1,PVRL2 |

**Table S4.** List of *ABs* involved in cancer-related GO terms for the HCC input set.

| Serial # | GO terms | *ABs* |
| --- | --- | --- |
| 1 | DNA replication | NUP98,FHIT,TIPIN,NFIC,CENPF,LOC100130009,HMGA1,Dnajc2,POLK |
| 2 | Negative regulation of phosphorylation | prkrip1,igfbp3,CDKN2A,INHBA |
| 3 | Negative regulation of myeloid leukocyte differentiation | NME2,NME1-NME2,Nme1,CTNNB1,INHBA |
| 4 | Vasculature development | amot,CXCL12,THY1,ANXA2P1,ANXA2P3,ANXA2,ROBO1,LOC642132,MIB1,BGN,id1,STAB2,CTNNB1 |
| 5 | DNA metabolic process | CDKN2A,CENPF,LOC100130009,HMGA1,PPIAL3,ppiA,LOC439953,SHFM1,TDRD1,NUP98,CYCS,TIPIN,FHIT,CUL4B,NFIC,FOS,PRKDC,LOC731751,Dnajc2,POLK |
| 6 | Spliceosome assembly | LOC100130109,SNRPEL1,SNRPE,SNRPD1,LOC100129492,Usp39,Celf1 |
| 7 | Chromatin organization | SMARCC1,tcf7l1,LOC399804,LOC100131044,LOC729686,Npm1,LOC729342,NPM1P21,LOC440577,BMI1,chd3,LOC100130009,HMGA1,ENY2,BRPF3,MAP3K12,LOC440926,h3f3a,LOC644914,LOC728914,H3f3b,PHF16,SETD2,SMARCD1,baz1b |
| 8 | Chromosome organization | SMARCC1,tcf7l1,NCAPH,LOC399804,LOC100131044,LOC729686,Npm1,LOC729342,NPM1P21,LOC440577,BMI1,chd3,CENPF,LOC100130009,HMGA1,ENY2,BRPF3,MAP3K12,LOC440926,h3f3a,LOC644914,LOC728914,H3f3b,DDX11,PHF16,SETD2,SMARCD1,baz1b,PRKDC,LOC731751 |
| 9 | Angiogenesis | CXCL12,THY1,ANXA2P1,ANXA2P3,ANXA2,ROBO1,LOC642132,id1,STAB2,CTNNB1 |
| 10 | Cell division | CCND1,TIPIN,SAC3D1,NCAPH,CETN2,TIMELESS,SSSCA1,CDKN2A,CENPF,cks1b,CFL1 |
| 11 | Blood vessel morphogenesis | amot,CXCL12,THY1,ANXA2P1,ANXA2P3,ANXA2,ROBO1,LOC642132,BGN,id1,STAB2,CTNNB1 |
| 12 | Blood vessel development | amot,CXCL12,THY1,ANXA2P1,ANXA2P3,ANXA2,ROBO1,LOC642132,MIB1,BGN,id1,STAB2,CTNNB1 |
| 13 | M phase of mitotic cell cycle | DDX11,TIPIN,SAC3D1,NCAPH,CETN2,TIMELESS,SSSCA1,CENPF,PES1,DCTN2 |
| 14 | Mitosis | DDX11,TIPIN,SAC3D1,NCAPH,CETN2,TIMELESS,SSSCA1,CENPF,PES1,DCTN2 |
| 15 | Interphase | CCND1,DDX11,TPD52L1,CDKN2A,CENPF,Dnajc2,INHBA |
| 16 | Interphase of mitotic cell cycle | CCND1,DDX11,TPD52L1,CDKN2A,CENPF,Dnajc2,INHBA |
| 17 | Cell cycle phase | CCND1,NCAPH,SSSCA1,CDKN2A,CENPF,TDRD1,PES1,DCTN2,INHBA,DDX11,TIPIN,SAC3D1,CETN2,TIMELESS,TPD52L1,Dnajc2 |
| 18 | Cell cycle process | CCND1,NCAPH,LOC399804,LOC100131044,LOC729686,Npm1,LOC729342,NPM1P21,LOC440577,SSSCA1,CDKN2A,PSMD4,CENPF,TDRD1,PSMD8,PES1,CTNNB1,DCTN2,INHBA,DDX11,TIPIN,SAC3D1,TIMELESS,CETN2,TPD52L1,Dnajc2 |
| 19 | Cell cycle | NCAPH,CDKN2A,PSMD4,PSMD8,PES1,CTNNB1,DCTN2,INHBA,DDX11,CETN2,TPD52L1,CCND1,LOC399804,LOC100131044,LOC729686,Npm1,LOC729342,NPM1P21,LOC440577,SSSCA1,CENPF,TDRD1,Ckap2,ERH,TIPIN,SAC3D1,TIMELESS,CUL4B,cks1b,tusc2,Dnajc2 |
| 20 | Mitotic cell cycle | CCND1,NCAPH,SSSCA1,CDKN2A,PSMD4,CENPF,PSMD8,PES1,DCTN2,INHBA,DDX11,TIPIN,SAC3D1,CETN2,TIMELESS,TPD52L1,Dnajc2 |
