# Supplementary material for: MiningABs: mining associated biomarkers across multi-connected gene expression datasets
Source: BMC Bioinformatics. 2014 Jun 8;15:173. doi: 10.1186/1471-2105-15-173 (PMC4068973; doi:10.1186/1471-2105-15-173)
Supplement: Additional file 3 — Example of different path lengths examined in this study. [file 1471-2105-15-173-S3.doc]

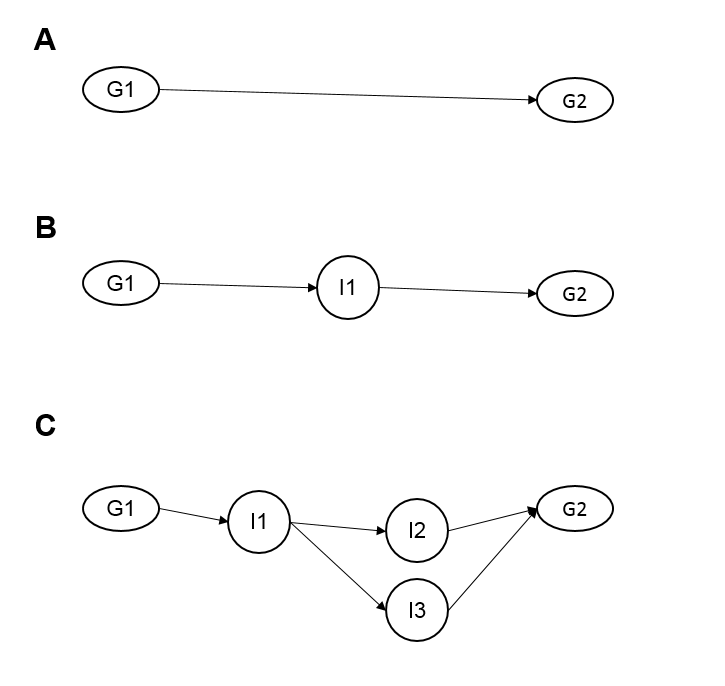


**Figure S1. Example of different path lengths examined in this study.** A) A length-1 path from gene G1 to G2. B) A length-2 path from gene G1 to G2 via intermediate gene I1. C) Two length-3 paths from gene G1 to G2 via intermediate gene I1-I2 and I1-I3. The ovals are ABs uploaded to IPA and the circles are intermediate genes as defined by IPA.
